# 5′-untranslated region sequences enhance plasmid-based protein production in *Sulfolobus acidocaldarius*

**DOI:** 10.3389/fmicb.2024.1443342

**Published:** 2024-11-25

**Authors:** Laura Kuschmierz, Alexander Wagner, Christian Schmerling, Tobias Busche, Jörn Kalinowski, Christopher Bräsen, Bettina Siebers

**Affiliations:** ^1^Molecular Enzyme Technology and Biochemistry (MEB), Environmental Microbiology and Biotechnology (EMB), Centre for Water and Environmental Research (CWE), Faculty of Chemistry, University of Duisburg-Essen, Essen, Germany; ^2^Microbial Genomics and Biotechnology, Center for Biotechnology (CeBiTec), Bielefeld University, Bielefeld, Germany

**Keywords:** Protein expression, Archaea, *Sulfolobus acidocaldarius*, 5’-untranslated region, Shine-Dalgarno

## Abstract

*Sulfolobus acidocaldarius*, a thermoacidophilic archaeon of the phylum Thermoproteota (former Crenarchaeota), is a widely used model organism for gene deletion studies and recombinant protein production. Previous research has demonstrated the efficacy of the *saci_2122* promoter (P_ara_), providing low basal activity and high pentose-dependent induction. However, the available expression vector does not include a 5′-terminal untranslated region (5’-UTR), a typical element found in bacterial expression vectors that usually enhances protein production in bacteria. To establish *S. acidocaldarius* as a production strain in biotechnology in the long term, it is intrinsically relevant to optimize its tools and capacities to increase production efficiencies. Here we show that protein production is increased by the integration of *S. acidocaldarius* 5’-UTRs into P_ara_ expression plasmids. Using the esterase Saci_1116 as a reporter protein, we observed a four-fold increase in soluble and active protein yield upon insertion of the *saci_1322* (*alba*) 5’-UTR. Screening of four additional 5’-UTRs from other highly abundant proteins (*thα*, *slaA*, *slaB, saci_0330*) revealed a consistent enhancement in target protein production. Additionally, site-directed mutagenesis of the Shine-Dalgarno (SD) motif within the *alba* 5’-UTR revealed its significance for protein synthesis. Ultimately, the *alba* 5’-UTR optimized expression vector improved the expression of various proteins, including six glycosyltransferases and one hydroxyacyl-CoA dehydratase from *S. acidocaldarius*, and a malto-oligosyltrehalose trehalohydrolase from *Saccharolobus solfataricus*, demonstrating its applicability. Our results show that the integration of SD-motif containing 5’-UTRs significantly enhanced plasmid-based protein production in *S. acidocaldarius*. This advancement in recombinant expression not only broadens the utility of *S. acidocaldarius* as an archaeal expression platform but also marks an important step toward potential biotechnological applications.

## 1 Introduction

The efficient production of properly folded, functional proteins of interest (POIs) is fundamental in both basic research and biotechnology. A range of microorganisms and gene expression vectors are available for protein production. Most commonly, yeast or bacteria are used for gene overexpression, with *Escherichia coli* standing out as the most commonly used bacterial expression host (Rosano and Ceccarelli, 2014). Despite continuous advancements in expression systems, challenges such as suboptimal codon usage and posttranslational modifications, protein misfolding, and the formation of insoluble aggregates persist, hindering successful gene overexpression (de Lise et al., 2023).

Selecting an appropriate expression host is crucial for successful POI production. Besides eukaryotic or bacterial strains, Archaea offer high potential as protein production platforms. Archaea are characterized by a mosaic nature, sharing cellular features with both Bacteria and Eukaryotes, while additionally exhibiting unique archaeal properties. As Bacteria, Archaea are unicellular, lack organelles, and possess similar DNA structures (e.g., operon structures, one circular chromosome, and plasmids). However, archaeal information processing (e.g., transcription and translation) and posttranslational modifications resemble simplified versions of eukaryotic processes (Lewis et al., 2021; Schocke et al., 2019; Esser et al., 2016; Jarrell et al., 2014; Eichler and Adams, 2005). Some archaeal proteins can be efficiently produced in *E. coli.* In particular, hyperthermophilic proteins can be easily purified by heat precipitation, which removes unwanted heat-unstable proteins of *E. coli* by denaturation, allowing their separation from the heat-stable, soluble target protein. However, the production of thermophilic and/or archaeal proteins is also often challenging using classical bacterial expression hosts (e.g., Martínez-Espinosa, 2019; Straub et al., 2018). In these cases, proper expression or protein folding and stability may depend on special cofactors, posttranslational modifications, or suitable expression conditions, such as temperature (Eichler and Adams, 2005; Martínez-Espinosa, 2019; Straub et al., 2018). Thus, expression in the native or a closely related organism was suggested as alternative for efficient protein synthesis (Straub et al., 2018).

With its established genetic system and genomic stability, the thermoacidophilic, aerobic archaeon *Sulfolobus acidocaldarius* (T_opt_ 70–75°C, pH_opt_ 2–3; Brock et al., 1972; Chen et al., 2005) offers a versatile platform for gene deletion studies and overexpression (de Lise et al., 2023; Wagner et al., 2012; Lewis et al., 2021). Inducible expression vectors utilizing sugar-inducible promoters (e.g., P_ara_ from *saci_2122*, P_xyl_ from *saci_1938* (both pentose inducible by L-/D-arabinose and D-xylose) or P_mal_ (maltose) from *saci_1165*) have been developed, featuring various N-or C-terminal protein tags (see van der Kolk et al., 2020 for an overview) for successful protein expression (e.g., Stracke et al., 2020; Zweerink et al., 2017). In a comparative promoter screening, P_ara_ was identified as the most efficient promoter, exhibiting the highest pentose-dependent induction with D-xylose and L-/D-arabinose and low basal activity (van der Kolk et al., 2020). While D-xylose and L-arabinose are metabolized by *S. acidocaldarius*, D-arabinose does not serve as a carbon source and can therefore act as an artificial inducer.

**Figure 1:**
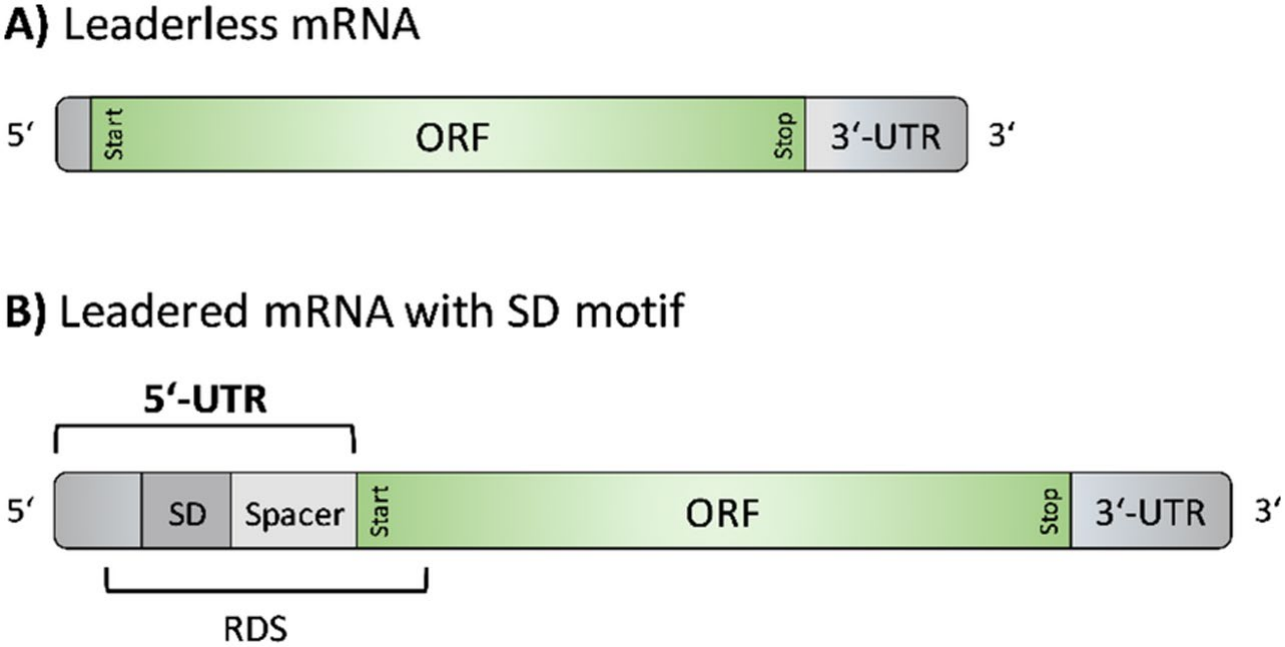
Structural elements of leaderless mRNAs and leadered mRNAs with a Shine-Dalgarno motif. **(A)** Leaderless mRNAs consist of an open reading frame (ORF), defined by a start and stop codon, followed by a 3′ untranslated region (UTR). Up to 10 nucleotides may optionally precede the ORF at the 5′ end (Brenneis et al., 2007). **(B)** Leadered mRNAs contain a 5’-UTR longer than 10 nucleotides, which may include a Shine-Dalgarno (SD) sequence. The region between the SD sequence and the start codon is known as spacer region. The ribosome docking site (RDS) includes the SD sequence, spacer and the first nucleotides of the coding region (Reeve et al., 2014; Chen et al., 1994).

In general, mRNAs can have UTRs at their 5′ and 3′ ends. Both are known to be involved in important regulatory processes, including transcription and translation initiation, transcript stability, mRNA secondary structure formation, and posttranscriptional regulation, e.g., through the action of RNA binding proteins or small regulatory RNAs (Ao et al., 2013; Brenneis and Soppa, 2009; Gomes-Filho et al., 2018; Ren et al., 2017). Notably, under the control of the native P_ara_ sequence, *S. acidocaldarius* generates leaderless gene transcripts, lacking a 5′-untranslated region (UTR) (Cohen et al., 2016). Leaderless mRNAs may either directly start with the initiation codon or possess up to five (Babski et al., 2016) or ten (Brenneis et al., 2007; Chen et al., 2015) 5′-terminal nucleotides upstream of the translation start codon (Figure 1). In contrast, leadered mRNAs carry a 5′- UTR, i.e., a non-coding regulatory sequence at the 5′-end of the transcript. 5’-UTRs may contain a Shine-Dalgarno (SD) sequence motif (Hering et al., 2009; Srivastava et al., 2016; Wurtzel et al., 2010). If present, the SD sequence [core sequence: GGAGGU (Shine and Dalgarno, 1975)] enables base pairing with the anti-Shine-Dalgarno sequence (aSD) at the 3′-end of the 16S rRNA of the small ribosomal subunit (Ma et al., 2002; Torarinsson et al., 2005), thereby supporting translation initiation. Based on the direct interaction of SD to aSD, the SD is referred to as ribosome binding site (RBS). However, in the process of translation initiation, the ribosome covers an mRNA region of approximately 30 nucleotides (nts), referred to as the ribosome docking site (RDS). This area includes the SD sequence, the translation start codon, an intermediate spacer region (of variable length and nucleotide composition), and the first nucleotides of the coding sequence (Reeve et al., 2014; Chen et al., 1994) (Figure 1).

The use of a 5’-UTR, including a preserved SD sequence, is common for many bacterial expression vectors, and in Bacteria the insertion, enlargement, or modification of the 5’-UTR (RBS) has been shown to increase plasmid-encoded protein production significantly (e.g., Liebeton et al., 2014; Vellanoweth and Rabinowitz, 1992; Volkenborn et al., 2020). In contrast, their utilization in archaeal expression plasmids remains less explored (e.g., Brenneis et al., 2007; Akinyemi et al., 2021). Notably, Wurtzel et al. (2010) reported that 5’-UTRs are overrepresented in Sulfolobales genes involved in translation, which are generally highly expressed. Thus, in this study, we systematically analyze the impact of 5’-UTR insertions of highly expressed genes into *S. acidocaldarius* expression plasmids on protein production, primarily utilizing esterase Saci_1116 and *β*-galactosidase LacS from *Saccharolobus solfataricus* (formerly *Sulfolobus solfataricus*) as reporters. Additionally, we employ the 5’-UTR-optimized expression vectors for the production of diverse proteins of different enzyme classes, including *S. acidocaldarius* glycosyltransferases as well as a hydroxyacyl-CoA dehydratase and *S. solfataricus* malto-oligosyltrehalose trehalohydrolase. Furthermore, we provide initial findings on the relevance of spacer nucleotides (located directly upstream of the translation start codon) and the SD sequence motif.

## 2 Materials and methods

### 2.1 Strains and growth conditions

The uracil auxotrophic mutant *S. acidocaldarius* MW001 (Wagner et al., 2012) was used as the expression host. The strain originates from *S. acidocaldarius* DSM639, but lacks 322 bp of *pyrE* (*saci_1597*), enabling genetic selection. Cultures were grown aerobically in Brock medium (Brock et al., 1972) at pH 3.0, 140 rpm, and 76°C (New Brunswick Innova 44 incubator, Eppendorf, Germany). Cell growth was monitored by turbidity measurements at 600 nm. *S. acidocaldarius* MW001 cultures were supplemented with 10 μg/mL uracil (min. 99%, Merck, Germany). Transformed clones of *S. acidocaldarius* MW001, carrying an expression plasmid with *pyrEF* from *Saccharolobus solfataricus*, were grown without the addition of uracil.

*Escherichia coli* DH5α (DSMZ 6897) was used for cloning and plasmid amplification. *E. coli* ER1821 (New England Biolabs, USA), harbouring pM.EsaBC4I, which encodes a DNA methylase and kanamycin resistance gene, was applied for the methylation of *S. acidocaldarius* expression plasmids. The strains were grown in Lysogeny broth (LB) medium (Carl Roth, Germany), supplemented with respective antibiotics, at 37°C (and 180 rpm for liquid cultures).

### 2.2 Plasmid construction

pBSaraFX-xxxUTR-Nt/Ct-SS plasmids (with xxx for the different UTRs tested) are originally based on pSVAaraFX-Nt/Ct-SS expression vectors (van der Kolk et al., 2020) (Supplementary Table S1). They possess the vector backbone of *Sulfolobus islandicus* plasmid pRN1 (Lipps, 2004) and contain basal shuttle vector elements such as origins of replication for *S. acidocaldarius* and *E. coli*, as well as *pyrEF* from *S. solfataricus* and an ampicillin resistance gene (*β*-lactamase) for selection in *S. acidocaldarius* or *E. coli*, respectively. The expression cassette for a gene of interest is flanked by different restriction sites and is composed of the pentose-inducible promoter of *saci_2122* (P_ara_) and *lacI*, *lacZ* as a reporter system enabling blue/white screening in *E. coli*. The vector encodes a Twin-Strep tag, enabling N-or C-terminal protein tagging (pSVAaraFX-NtSS or-CtSS; van der Kolk et al., 2020). “FX” indicates that the FX cloning strategy can be applied (class IIS restriction enzyme *Sap*I; Geertsma and Dutzler, 2011).

#### 2.2.1 Integration of different 5’-UTR sequences into *saci_1116* expression plasmid

In this study, the pSVAaraFX-*saci_1116*-CtSS expression plasmid was expanded by the insertion of different 5′-untranslated region sequences that were integrated directly upstream of the translation start codon of the reporter gene *saci_1116* (Supplementary Figures S1, S2). The insertion of a 5’-UTR was achieved using standard cloning procedures: modified promoter-5’-UTR fragments were PCR amplified with a fwd-primer (UTR-fwd, Supplementary Table S2), that bound to the vector backbone upstream of the *saci_2122* promoter, in combination with a rev-primer containing the desired 5’-UTR sequence and a *Nco*I restriction site that bound upstream of the translation start codon (Supplementary Table S2). pSVAaraFX-CtSS served as PCR template. The resulting PCR product consisted of a fragment of the vector backbone, carrying a *Sac*II restriction site, P_ara_, and the newly integrated 5’-UTR sequence with a *Nco*I restriction site. The fragment was cloned into pSVAaraFX-*saci_1116*-CtSS by restriction with *Sac*II and *Nco*I (NEB) followed by ligation (T4 DNA ligase, NEB). The resulting *saci_1116* expression plasmid, containing a 5’-UTR, was named pBSaraFX-xxxUTR-*saci_1116*-CtSS. Successful cloning was confirmed by sequencing (LGC genomics, Germany).

#### 2.2.2 Generation of *lacS* expression constructs with different 5’-UTR sequences

The series of expression plasmids for *saci_1116*, each containing distinct 5’-UTR sequences, served as the basis for the development of corresponding *lacS* expression plasmids. Amplification of *lacS* from *S. solfataricus* (SSO3019) was conducted using primers *lacS*-SSO-*Nco*I-fwd and *lacS*-SSO-*Xho*I-rev (Supplementary Table S2). Subsequently, the resulting PCR product and the pBSaraFX-xxxUTR-*saci_1116*-CtSS expression plasmids (Supplementary Table S1) underwent restriction enzyme digestion with *Nco*I and *Xho*I. This process facilitated the excision of *saci_1116* from the xxxUTR-plasmid backbone, excluding it from subsequent cloning steps through agarose gel extraction of the plasmid fragment. The *Nco*I-*Xho*I digested pBSaraFX-xxxUTR-CtSS fragment and *lacS* were ligated. Verification of the resultant plasmid set, designated as pBSaraFX-xxxUTR-*lacS*-CtSS, was conducted through sequencing. Subsequently, this plasmid set was utilized to investigate LacS production in *S. acidocaldarius*.

#### 2.2.3 Site-directed mutagenesis of *alba* 5’-UTR

To assess the impact of -2 and -1 nucleotides, located directly upstream of the translation start codon within the *alba* 5’-UTR, on gene expression, we employed site-directed mutagenesis. Plasmid pBSaraFX-*alba*UTR-*saci_1116*-CtSS served as the template for modifications (Supplementary Table S1). Each primer contained the desired mutation and was complementary to the other (Supplementary Table S2). To prevent the formation of primer homodimers in the reaction mixture, DNA amplification was performed in single-primer reactions, following the protocol outlined by Edelheit et al., 2009. Q5^®^ High-Fidelity DNA Polymerase and its recommended reaction buffer (NEB) were utilized for the amplification process.

Correspondingly, the SD sequence motif within the *alba* 5’-UTR was modified by site-directed mutagenesis, as described by Edelheit et al. (2009) (Supplementary Table S2) using pBSaraFX-*alba*UTR-*saci_1116*-CtSS as template for the “noSD” and “8SDmut” variants of *alba* 5’-UTR (Supplementary Tables S1, S2). The “8SDins” variant, designated as pBSaraFX-*alba*UTR-8SDins-*saci_1116*-CtSS, was constructed via restriction and ligation-dependent cloning. Initially, PCR was conducted using primers SD-fwd and 8SD-ins-rev with pSVAaraFX-CtSS serving as the template (Supplementary Tables S1, S2). Subsequently, the resulting PCR product and the vector pSVAaraFX-*saci_1116*-CtSS underwent restriction using *Sac*II and *Nco*I, followed by ligation.

#### 2.2.4 Generation of cloning vectors pBSaraFX-*alba*UTR-CtSS and -NtSS

Generally, *S. acidocaldarius* expression vectors contain *lacI* and *lacZ* in the expression cassette for blue/white screening in *E. coli*. To enable this feature to be used in the cloning process of *alba* 5’-UTR containing plasmids, two *alba* 5’-UTR cloning vectors with inserted *lacI*, *lacZ*, and an N-or C-terminal Twin-Strep tag were generated. pBSaraFX-*alba*UTR-NtSS (N-terminal Twin-Strep tag) was generated by the insertion of the original *alba* (*saci_1322*) 5’-UTR (5′ GATAGGTGGTTTAA 3′) directly upstream of the gene expression cassette start codon into pSVAaraFX-NtSS by overlap PCR using the primer pair *saci_1322*-UTR-NtSS-fwd and -rev (Supplementary Table S2). To obtain the cloning vector pBSaraFX-*alba*UTR-CtSS (C-terminal Twin-Strep tag), the *saci_1116* expression plasmid pBSaraFX-*alba*UTR-*saci_1116*-CtSS as well as the vector pSVAaraFX-CtSS, containing *lacI* and *lacZ,* were restricted with *Nco*I and *Xho*I. The obtained vector backbone and the *lacI-lacZ* fragment were ligated, resulting in cloning vector pBSaraFX-*alba*UTR-CtSS (containing the modified *alba* 5’-UTR: 5’ GATAGGTGGTTTCC 3′).

#### 2.2.5 Cloning of gene expression plasmids for different target proteins

Genes of interest, namely *saci_1904*, *saci_1909*, *saci_1911*, *saci_1915*, *saci_1916*, *saci_1921*, *saci_2033*, and *saci_1085* from *S. acidocaldarius* and SSO3019 (*lacS*) and SSO2093 (*treZ*) from *S. solfataricus* were cloned into pSVAaraFX-NtSS and/or-CtSS as well as pBSaraFX-*alba*UTR-NtSS and/or-CtSS expression vectors (Supplementary Table S1). All cloning primers and the used restriction sites are included in Supplementary Table S2.

### 2.3 Transformation of *S. acidocaldarius* MW001

Competent cells were prepared according to Wagner et al. (2012), and transformed with 300–500 ng of methylated plasmid DNA using a Gene Pulser Xcell (Bio-Rad, Germany) at 2,000 V, 600 *Ω* and 25 μF in 1 mm cuvettes. Cell recovery was performed in Brock medium (pH 5.0), supplemented with 0.1% (*w/v*) N-Z-amine (casein enzymatic hydrolysate N-Z-Amine^®^ AS, Merck, Germany), for up to 4 hours at 76°C in a heat block (speed of 300 rpm; ThermoMixer F1.5, Eppendorf, Germany) (Wagner et al., 2014). Cells were plated on Gelrite-Brock plates with 0.6% (*w/v*) Gelrite^®^ (Gellan Gum: K9A-40, Serva Electrophoresis, Heidelberg, Germany), 0.1% (*w/v*) N-Z-amine and 0.2% (*w/v*) dextrin (pure, Carl Roth, Germany), lacking uracil. Plates were incubated in a closed metal box for 5–6 d at 76°C.

### 2.4 *S. acidocaldarius* MW001 expression cultures

After transformation of *S. acidocaldarius* MW001 and incubation of cells on Gelrite-Brock plates, clones were transferred to liquid Brock medium (0.1% (*w/v*) N-Z-amine (Merck, Germany) and 0.2% (*w/v*) dextrin (Carl Roth, Germany), pH 3.0), and cultures were grown to the late log growth phase (OD_600nm_ 0.9–1.2) at 140 rpm and 76°C. The presence of the expression plasmid was verified by colony PCR using gene-specific primer pairs (Supplementary Table S2). Cell cultures, carrying an expression plasmid with SSO*pyrEF*, were used to inoculate pre-cultures for *S. acidocaldarius* expression. Pre-cultures were cultivated in Brock medium supplemented with 0.1% (*w/v*) N-Z-amine (Merck, Germany) and 0.2% (*w/v*) dextrin (Carl Roth, Germany) without uracil until the log growth phase. Expression cultures were inoculated to an OD_600nm_ of 0.05–0.1, and 0.1% (*w/v*) N-Z-amine as well as 0.3% (*w/v*) D-xylose (min. 99%, Carl Roth, Germany) were added to the medium. Expression cultures were grown at 140 rpm and 76°C (New Brunswick Innova 44 incubator, Eppendorf, Germany). Cells were harvested by centrifugation (7,000 xg, 4°C, 20 min) at OD_600nm_ values of 0.6 or 0.9–1.2, as stated in the results section. Cell pellets were stored at −80°C until further use.

### 2.5 Sodium dodecyl sulfate-polyacrylamide gel electrophoresis (SDS-PAGE) and immunoblotting of *S. acidocaldarius* cell lysate samples

For the comparison of protein expression in presence and absence of a certain 5’-UTR sequence, *S. acidocaldarius* cell samples were adjusted to a theoretical OD_600nm_ of 10 by resuspension of cells in appropriate volumes of 50 mM TRIS/HCl pH 7.5 with 0.5% (v/v) Triton X-100 (Gerbu, Germany). Samples of protein purification were applied to SDS-PAGE. 5x SDS-PAGE loading dye (125 mM TRIS/HCl pH 6.8, 4% (w/v) SDS, 20% (v/v) glycerol, 10% (v/v) *β*-mercaptoethanol, 0.01% (w/v) Bromophenol Blue) was added and samples were heated for 5 min at 99°C. 5 μL of PageRuler Unstained or PageRuler (Plus) Prestained Protein Ladder (Thermo Fisher Scientific, USA) and 15 or 20 μL of each cell lysate sample (approximately 30 μg of protein) were applied to SDS-PAGE. Gels were either stained by Coomassie Brilliant Blue staining (0.05% (*w/v*) Coomassie Brilliant Blue G-250 (Merck, Germany), 40% (*v/v*) ethanol, 10% (*v/v*) acetic acid) and imaged using the Molecular Imager Gel Doc XR System and the Quantity One Software Package (Bio-Rad, Germany), or they were used for immunodetection. Proteins were transferred on a PVDF membrane (Roti-PVDF, pore size 0.45 μm, Carl Roth, Germany) using blotting buffer (50 mM TRIS, 40 mM glycine, 0.1% (*w/v*) SDS, 20% (*v/v*) EtOH (p.a.)) and the Bio-Rad trans-blot^®^ TurboTM Trans System (25 V, 0.5–1 A, 25 min; Bio-Rad, Germany). Afterwards, the membrane was blocked with 0.2% (*w/v*) Tropix^®^ I-BLOCK (Applied Biosystems, Thermo Fisher Scientific, USA) in TBS-T buffer (20 mM TRIS–HCl pH 7.5, 500 mM NaCl, 0.05% (*v/v*) Tween 20) at 4°C overnight. The next day, the membranes were floated in a solution of 1:50,000 Strep-Tactin HRP conjugate (IBA Lifesciences, Germany) with 0.1% (*w/v*) I-BLOCK in TBS-T buffer and incubated while shaking at RT for 1–2 h. The membrane was washed twice using 0.1% (*w/v*) I-BLOCK in TBS-T buffer for 10 min each, followed by two washing steps using TBS-T and TBS buffer, respectively. Clarity Western ECL Substrate (Bio-Rad, Germany) and the VersaDoc MP-4000 imaging system (Bio-Rad, Germany) were used for immunodetection and documentation.

### 2.6 Bicinchoninic acid (BCA) protein assay of *S. acidocaldarius* cell lysates

*S. acidocaldarius* cell suspensions were adjusted to a theoretical OD_600nm_ of 5 by resuspension of cells in 50 mM TRIS/HCl pH 7.5 with 0.5% (v/v) Triton X-100 (Gerbu, Germany). Cells were lysed by sonication [ultrasonic processor UP 200 s (Hielscher Ultrasonics, Germany)] with a cycle of 0.5 s^−1^ and an amplitude of 50% for 1 min on ice. Protein concentrations in cell lysate samples were determined using the bicinchoninic acid (BCA) assay (Uptima BC assay protein quantification kit, France). Cell lysates (25 μL) and bovine serum albumin (BSA) protein standards (0–1 mg/mL BSA (Merck, Germany) in resuspension buffer) were transferred in the wells of a 96-well microtiter plate (MTP; flat bottom, polystyrene, Sarstedt, Germany) and mixed with 200 μL BCA solution each. For each biological replicate three technical replicates were analyzed. After 3 h of incubation at room temperature absorption at 562 nm was determined in a Tecan plate reader (Tecan Infinite^®^ M200, NanoQuant PlateTM; Tecan, Switzerland).

### 2.7 Esterase activity assay (Saci_1116)

To determine the esterase activity of Saci_1116 in cell lysates of *S. acidocaldarius* cultures, *para*-nitrophenyl acetate (*p*NPA, Merck, Germany) was used as a substrate (Sobek and Görisch, 1988, 1989). 175 μL of 50 mM TRIS/HCl (pH 7.5) and 5 μL of 10-fold diluted *S. acidocaldarius* cell lysate sample (diluted with reaction buffer) were pipetted into the wells of a 96-well MTP (flat bottom, polystyrene, Sarstedt, Germany) and pre-warmed in the Tecan plate reader at 42°C for 10 min (Tecan Infinite^®^ M200, NanoQuant PlateTM; Tecan, Switzerland). Afterward, 20 μL of 10 mM *p*NPA (dissolved in acetonitrile) were added to the wells by automatic dispersion, yielding a substrate concentration of 1 mM *p*NPA in the assay. Measurements were performed in 60 s cycles at 410 nm for 1 h, following the formation of *p*NP (4-nitrophenol). *p*NP (Merck, Germany) calibration curves were conducted under the same conditions. Experiments were performed in three biological replicates and for each biological replicate three technical replicates were analysed. The specific activity for biological replicates was calculated and displayed as data points in the bar charts. Enzyme activity measurements of purified esterase Saci_1116-CtSS were performed under the same assay conditions, using 40 and 20 ng of pure protein. The initial reaction velocities were used for the calculation of activities. *p*NP production was calculated using the established *p*NP calibration curve. Specific enzyme activities (U/mg of protein) were determined by the protein concentration obtained in the BCA assay.

### 2.8 *β*-galactosidase activity assay (LacS)

Cells of *S. acidocaldarius* expression cultures were resuspended in 50 mM sodium phosphate buffer (pH 6.5) supplemented with 50 mM NaCl and disrupted using prefilled 0.1 mm Glass Bead tubes and the Precellys 24 tissue homogenizer (Bertin technologies, France). The cell lysis process involved three cycles of homogenization at 6.500 xg for 20 s each, with intermittent cooling on ice for 1 min between cycles. Subsequently, cell lysates were centrifuged at 21,428 xg at 4°C for 40 min to obtain the supernatant, the crude extract, which was next utilized for protein concentration determination. Protein concentration was assessed using the Bradford protein assay (QuickStart™, Bio-Rad, Germany) with BSA (Sigma-Aldrich, USA) as protein standard (Zor and Selinger, 1996). LacS activity in crude extracts was determined using a continuous assay in the BioTek Synergy H1 microplate reader with 96-well microtiter plates (flat bottom, polystyrene, Thermo Fisher Scientific, USA). The wells of a 96-well MTP were filled with 177.5 μL of 50 mM sodium phosphate buffer (pH 6.5) and 2.5 μL of crude extract and pre-warmed in the reader at 70°C for 20 min. Then, 20 μL of 10 mM *para-*nitrophenyl β-D-galactopyranoside (dissolved in buffer) were automatically dispensed into the wells, resulting in a final substrate concentration of 1 mM *p*NPG in the assay. The plate was shaken for 5 s, and measurements were performed in 60 s cycles at 410 nm for 30 min to monitor the formation of *p*NP (4-nitrophenol). Calibration curves for *p*NP were prepared under the same conditions. For each biological replicate three technical replicates were analyzed. Finally, the initial linear range of *p*NP formation following the enzymatic reaction (8 min) was utilized to calculate specific enzyme activity.

### 2.9 Purification of Twin-Strep-tagged proteins from *S. acidocaldarius* crude extracts

Purification was performed according to the manufacturer’s instructions (IBA Lifesciences, Germany). Briefly, *S. acidocaldarius* cell suspensions were adjusted to a theoretical OD_600nm_ of 5 by resuspension in buffer W (100 mM TRIS/HCl pH 8.0, 150 mM NaCl). Cells were disrupted by sonication (UP 200 s, Hielscher Ultrasonics, Germany) with a cycle of 0.5 s^−1^ and an amplitude of 60% three times for 5 min each on ice (cooling pauses of 1 min each) and cell lysates were centrifuged at 16,000 xg and 4°C for 40 min to remove cell debris. Supernatants were filtered (0.45 μm polyvinylidene fluoride membrane, Carl Roth, Germany) and Twin-Strep tagged proteins of interest were purified from crude extracts (soluble fraction) using the Strep-Tactin^®^XT Superflow^®^ Kit (IBA Lifesciences, Germany). Purification was performed according to the manufacturer’s instructions, using buffer BXT (100 mM TRIS/HCl pH 8.0, 150 mM NaCl, 1 mM EDTA, 50 mM biotin) as elution buffer. Protein purity was analyzed by SDS-PAGE and the protein concentration was determined using the Bradford assay (QuickStart™, Bio-Rad, Germany) with BSA as the protein standard (Zor and Selinger, 1996).

## 3 Results

### 3.1 Influence of the *alba* 5’-UTR on the plasmid-encoded production of esterase Saci_1116

In earlier studies, the P_ara_ promoter (*saci_2122*) has demonstrated its effectiveness in enabling protein production in *S. acidocaldarius*, with pentose-dependent induction and low basal activity (van der Kolk et al., 2020). Notably, RNA sequencing data (Cohen et al., 2016; https://exploration.weizmann.ac.il/TCOL/) indicate that this promoter sequence leads to the generation of leaderless gene transcripts. This prompted our investigation into whether the incorporation of a 5’-UTR (RBS) could enhance plasmid-based protein production. To assess the impact of a 5’-UTR on protein yield, we utilized the 5’-UTR sequence of the Alba encoding gene *saci_1322* (*alba*) from *S. acidocaldarius*. Alba (Acetylation Lowers Binding Affinity) (Laurens et al., 2012) is a well-conserved archaeal double-stranded DNA/RNA binding protein (Wardleworth et al., 2002; Cajili and Prieto, 2022) and it was already reported to be highly abundant in *Sulfolobus* [4.8% of cellular protein in *Sulfolobus shibatae* (Mai et al., 1998)]. In agreement, available full proteome data from *S. acidocaldarius* identified *Alba* as the most abundant cellular protein under different growth conditions (label-free quantification intensity, 0.2% (w/v) NZA and 0.2% (w/v) D-xylose; Schmerling et al., 2024a). The *alba* transcript comprises a 14-nucleotide long 5’-UTR (5’ GAU**AGGUG**GUUUAA 3′) (Cohen et al., 2016; Schmerling et al., 2024a). Based on previous literature on *Sulfolobus* SD consensus as well as *S. acidocaldarius* aSD sequence (e.g., Tolstrup et al., 2000; Schmitt et al., 2020), we identified a 5-nucleotide long SD sequence motif (bold), followed by a 6-nucleotide long spacer in *alba* 5’-UTR. To evaluate the impact of this 5’-UTR on protein production, we selected the esterase Saci_1116 as reporter protein. The enzyme was previously characterized (Sobek and Görisch, 1988, 1989), and was used as a model protein in the optimization of the *S. acidocaldarius* expression system before (van der Kolk et al., 2020). We used existing expression plasmids containing the P_ara_ promoter and encoding *saci_1116*, with either an N-or C-terminal Twin-Strep tag (NtSS or CtSS, respectively; Supplementary Table S1), as templates for inserting the *alba* 5’-UTR and as reference for expression analysis. The *alba* 5’-UTR was inserted upstream of the reporter gene start codon (“ATG”; Supplementary Figure S1), resulting in the generation of *S. acidocaldarius* expression plasmids termed pBSaraFX-***alba*UTR**-*saci_1116*-NtSS or-CtSS, respectively (Supplementary Figures S1, S2; Supplementary Table S1).

**Figure 2:**
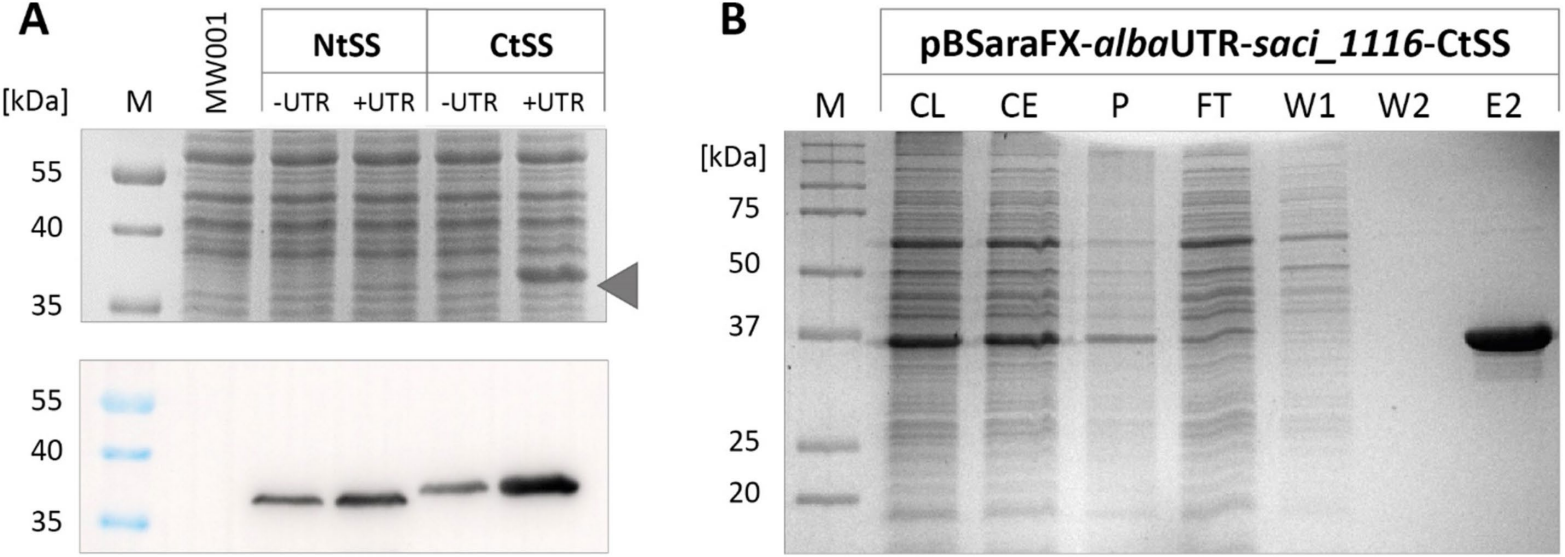
Effect of *alba* 5’-UTR on esterase Saci_1116 production in *S. acidocaldarius*. **(A)** Coomassie-stained SDS-PAGE and immunoblot of Twin-Strep-tagged Saci_1116 in cell lysates from *S. acidocaldarius* expressing *saci_1116* without and with *alba* 5’-UTR, and with N-terminal (Nt) or C-terminal (Ct) Twin-Strep tag (SS). The arrow indicates the height of N-terminal tagged Saci_1116 bands in the Coomassie-stained SDS-PAGE. *S. acidocaldarius* MW001 without plasmid served as reference (MW001). For all cell samples, OD_600nm_ was adjusted to 10, and 15 μL of each lysate were applied to SDS-PAGE. **(B)** SDS-PAGE of Saci_1116-CtSS purification from crude extract of *S. acidocaldarius* MW001 pBSaraFX-*alba*UTR-*saci_1116*-CtSS via affinity chromatography. M: marker, CL: cell lysate; CE: crude extract (after centrifugation); P: cell pellet and debris after lysis; FT: flow-through; W: wash fraction; E: elution, 3 μg protein. Theoretical molecular weight of Saci_1116-SS: 37 kDa.

Insertion of the *alba* 5’-UTR into the *saci_1116*-CtSS expression plasmid markedly enhanced reporter protein yield, as evidenced by SDS-PAGE and immunodetection analysis (Figure 2A). In line with previous observations (van der Kolk et al., 2020), the C-terminal tag yielded higher Saci_1116 production in contrast to the N-terminal protein tag. Using the C-terminal Twin-Strep tag, Saci_1116 was purified from the soluble protein fraction from the UTR expression culture (Figure 2B), and protein quantification revealed a four-fold increase in reporter protein yield using the *alba* 5’-UTR modified expression plasmid compared to the vector lacking the 5’-UTR (Supplementary Figure S3). The purified esterase Saci_1116-CtSS exhibited a specific activity of 124.5 U/mg using *p*NP-acetate as a substrate at 42°C. In total, the incorporation of the *alba* 5’-UTR into the *saci_1116*-CtSS expression plasmid significantly enhanced the production of soluble, active esterase reporter protein.

### 3.2 Effect of 5’-UTR insertion on promoter activity

To assess the impact of *alba* 5’-UTR insertion on the functionality and selective induction of the pentose-inducible promoter (*saci_2122* promoter P_ara_), we investigated protein production under the influence of different inducing and non-inducing sugars (Supplementary Figure S4). While *S. acidocaldarius* metabolizes the inducing pentoses D-xylose and L-arabinose, D-arabinose is not utilized as carbon source and hence acts as an artificial inducer and was therefore applied at a lower concentration. Our findings demonstrate that the functionality of the pentose-inducible promoter remained unaffected by the insertion of the *alba* 5’-UTR into the expression construct (Supplementary Figure S4). In agreement with previous studies, gene expression was induced by the addition of pentoses, while minimal basal expression was observed with sucrose, the disaccharide comprising D-glucose and D-fructose (Supplementary Figure S4; van der Kolk et al., 2020). Thus, *alba* 5’-UTR insertion resulted in a profound increase in reporter protein levels, while maintaining P_ara_’s inducibility and pentose specificity.

### 3.3 Maintenance of cloning sites—influence of −2 and −1 nucleotide identities on reporter protein production

**Figure 3:**
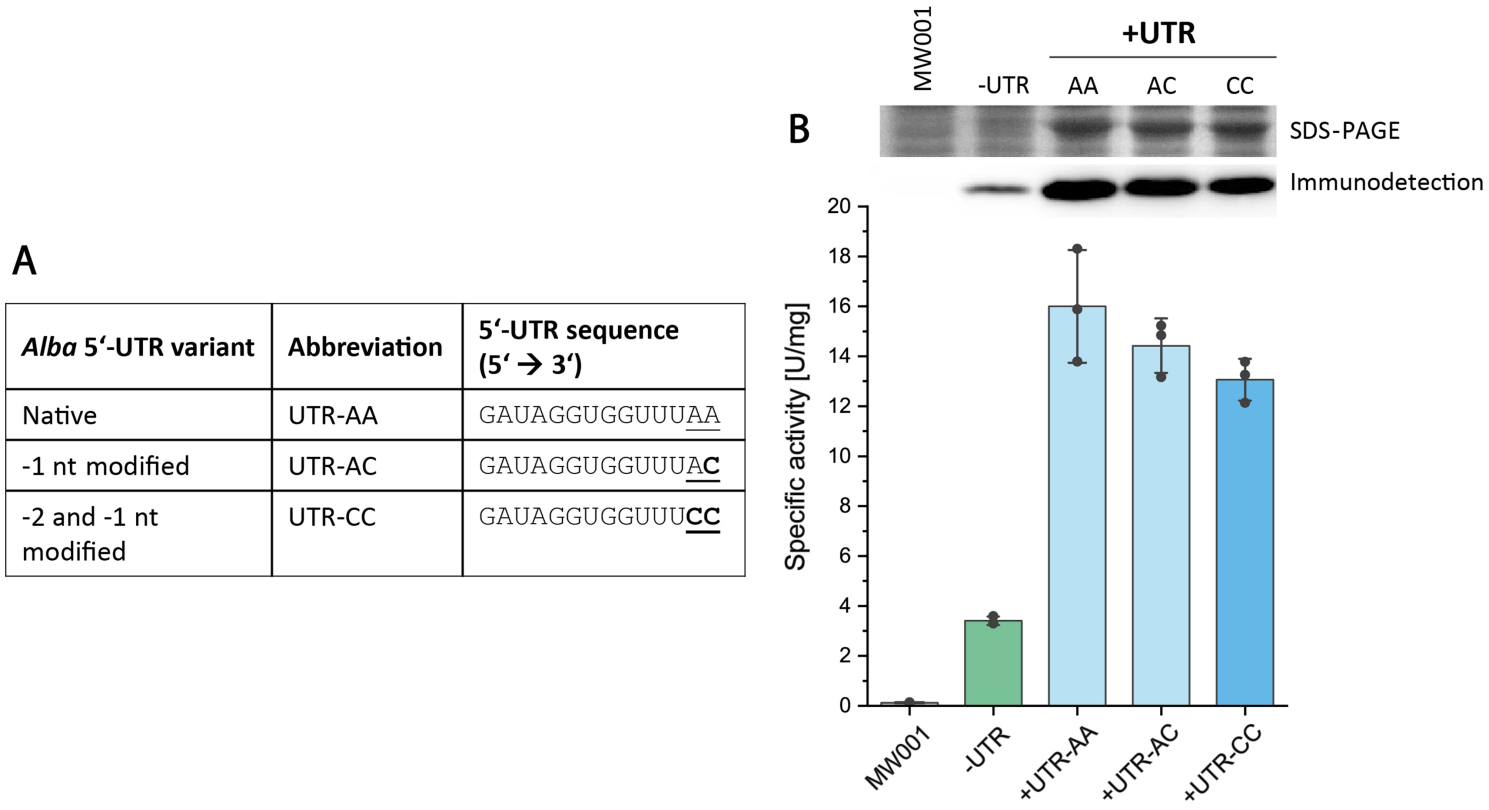
Influence of-2 and -1 nucleotide identities in the *alba* 5’-UTR on esterase production. **(A)** Overview of different *alba* 5’-UTR variants tested. **(B)** Expression analysis of Saci_1116-CtSS using constructs either without a 5’-UTR (-UTR) or with the *alba* 5’-UTR variants. Esterase activity was quantified in cell lysates using a continuous assay with *p*NP-acetate as substrate, monitoring *p*NP formation at 410 nm and 42°C. Error bars represent the standard deviation from three biological replicates (*n* = 3), shown as data points. Image sections of the respective SDS-PAGE (upper row) and Saci_1116-CtSS immunodetection (lower row) are provided (uncropped images in Supplementary Figure S5). Cultures were harvested at OD_600nm_ values of 0.6 (log phase). *S. acidocaldarius* MW001 without plasmid served as a reference (MW001).

Expression vectors should facilitate the seamless integration of genes of interest (GOIs) through the presence of suitable restriction sites for restriction-and ligation-dependent cloning. In the pSVAara expression vectors, a *Nco*I restriction site (5’ C’CATGG 3′) serves as the upstream cloning site for GOI integration. For vectors featuring N-terminal protein tags, this *Nco*I site is positioned downstream of the tag-encoding nucleotide sequence. Thus, the integration of a 5’-UTR upstream of the N-terminal tag-coding sequence does not affect the *Nco*I cloning site (Supplementary Figure S1). However, in vectors with C-terminal protein tags, the direct insertion of the native *alba* 5’-UTR sequence (5′ GATAGGTGGTTTAA 3′) upstream of the translation start codon (“ATG”) would disrupt the *Nco*I restriction site used for GOI integration, which simultaneously encodes the translation start codon in C-terminal tag vectors (*Nco*I site: 5’ C’C**ATG**G 3′, start codon in bold) (Supplementary Figure S1). Therefore, for convenient use, the two nucleotides located at the 3′-end of the 5’-UTR, immediately upstream of the start codon, hereafter referred to as −2 and − 1 nucleotides, were modified to preserve the *Nco*I restriction site in C-terminal protein tag vectors. Specifically, the identities of the −2 and −1 nucleotides were changed from “AA” to “CC,” while maintaining the overall length of the *alba* 5’-UTR. We assessed the impact of these nucleotide changes (i.e., from “AA” to “AC” or “CC”) on reporter protein production through enzyme activity assays, SDS-PAGE, and immunodetection (Figure 3).

Similar values of specific esterase activities were obtained in the cell lysates of respective *S. acidocaldarius* expression cultures for all tested −2 and −1 nucleotide identities (“AA,” “AC” and “CC”) (Figure 3, Supplementary Table S3). SDS-PAGE analysis and esterase immunodetection confirmed the results of activity measurements (Supplementary Figure S5). In comparison to expression conditions lacking a 5’-UTR, constituting 2.5% of the total cell protein, the insertion of the *alba* 5’-UTR variants resulted in a 3.9–4.7-fold increase in esterase activity, with the reporter enzyme constituting 10–13% of cellular protein (Supplementary Table S3). This share was calculated by comparing the specific activities in cell lysates to the specific activity of purified esterase (124.5 U/mg).

### 3.4 Screening of different UTR candidates for efficient reporter protein production

**Figure 4:**
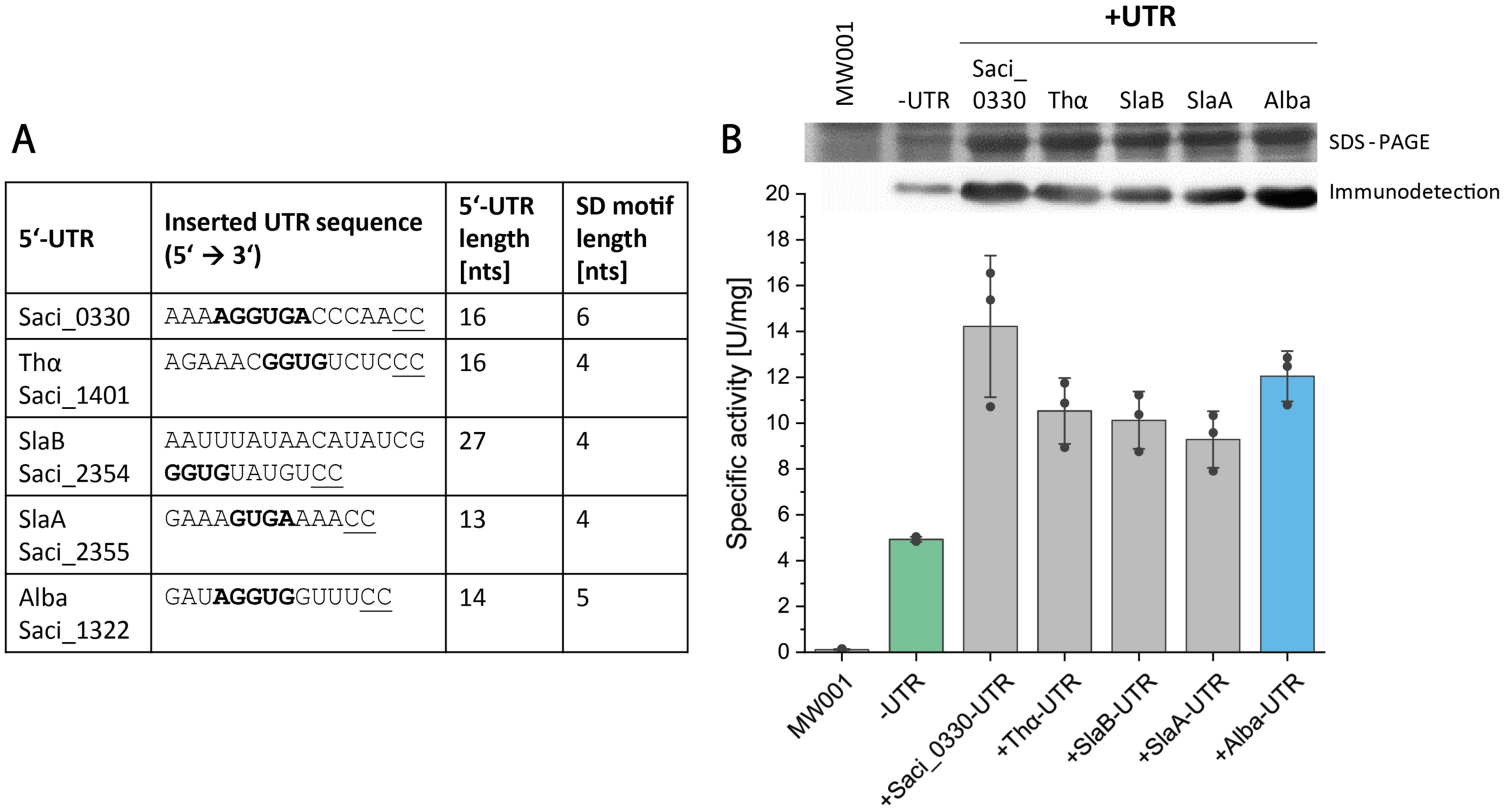
Screening of five 5’-UTR sequences for Saci_1116-CtSS production. **(A)** Overview of tested 5’-UTR sequences, including gene IDs, 5’-UTR sequences with their respective SD motifs (bold), UTR lengths, and SD motif lengths. **(B)** Saci_1116-CtSS production was analyzed using constructs without (-UTR) or with a 5’-UTR (+UTR). The 5’-UTR sequences from *saci_0330*, *saci_1401* (Thα), *saci_2354* (SlaB), *saci_2355* (SlaA), and *saci_1322* (Alba) were inserted upstream of the *saci_1116*-CtSS start codon in the expression plasmid. Specific esterase activity was determined in cell lysates using the continuous *p*NPA assay. Error bars represent the standard deviation from three biological replicates (*n* = 3), shown as data points. Sections of the respective SDS-PAGE of cell lysates (upper row) and immunodetection (lower row) are shown (uncropped images in Supplementary Figure S6). Cultures were harvested at OD_600nm_ values of 0.8–1.2 (log phase). *S. acidocaldarius* MW001 without plasmid served as a reference (MW001).

In the next step, we sought to ascertain whether the efficacy of the expression system could be augmented by utilizing alternative 5’-UTR sequences. Leveraging literature and available proteomic data, we identified proteins of notable abundance in *S. acidocaldarius* and extracted their respective 5’-UTR sequences from RNA sequencing data (Weizmann Exploration, https://exploration.weizmann.ac.il/TCOL/; Cohen et al., 2016). Four 5’-UTR sequences, derived from genes *saci_0330* (encoding a hypothetical protein), *saci_1401* (thermosome *α* subunit), *saci_2355* (surface-layer protein A), and *saci_2354* (S-layer protein B), were chosen for comparative analysis alongside the *alba* 5’-UTR (Figure 4A).

The 5’-UTR sequence of *saci_0330* corresponds to the transcript leader sequence of the operon *saci_0330–0333*, with *saci_0331* annotated to encode a pyridine nucleotide-disulphide oxidoreductase, while the others are annotated as conserved proteins of unknown function (Chen et al., 2005; Esser et al., 2011). *Saci_2355* and *saci_2354*, encoding S-layer proteins A and B, with their respective 5’-UTR sequences are encoded in a single operon (*slaAB*) on the complementary DNA strand (Veith et al., 2009). However, in addition to the promoter upstream of SlaA, available transcriptome data revealed a second promoter and a weak 5’-UTR signal upstream of *slaB*, consistent with RNA sequencing data (Cohen et al., 2016). The 5’-UTR of *saci_1401*, the gene encoding the thermosome α subunit (*thα*), a major chaperone complex in Sulfolobales (Chaston et al., 2016; Baes et al., 2020), and the *alba* 5’-UTR originate from single gene transcripts (Cohen et al., 2016). Based on the *Sulfolobus* SD consensus sequence (GGUGA; Tolstrup et al., 2000) and the further potential of base-pairing between 5’-UTR sequence and aSD sequence, we proposed the presence of a 6- and 5-nucleotide long SD motif in the 5’-UTRs of *saci_0330* and *alba,* respectively. The other three UTR sequences (*thα*, *slaB, slaA*) included SD motifs of 4 nts in length (Figure 4A). As before, the *Nco*I restriction and cloning site (5’ C’C**ATG**G 3′) was maintained, i.e., the nucleotide identities at positions -2 and -1 (relative to the translation start codon) were changed to “CC” for all 5’-UTR sequences tested, while preserving the overall length of each 5’-UTR (Figure 4A; Supplementary Table S4). Screening was conducted as previously described using esterase Saci_1116-CtSS as reporter protein, and its production was evaluated by specific esterase activity, SDS-PAGE, and immunodetection (Figure 4).

The 5’-UTR screening revealed significantly increased reporter protein activity in the presence of all tested 5’-UTR sequences compared to the absence of a 5’-UTR (Figure 4). Activity-based results were confirmed through SDS-PAGE analysis and esterase immunodetection (Figure 4B; Supplementary Figure S6). Integration of different 5’-UTR sequences increased the amount of reporter protein to approximately 7.5–11% of the total protein compared to 3% without an inserted 5’-UTR (Supplementary Table S5). On average, the 5’-UTRs of *saci_0330* and *alba* exhibited the highest production yield of active esterase, with a lower standard deviation for *alba* 5’-UTR (Figure 4B).

In addition to the esterase, we also investigated the impact of these 5’-UTRs on another reporter protein, *β*-galactosidase LacS from *S. solfataricus* (Supplementary Figures S7, S8) (Pisani et al., 1990). LacS was commonly employed as a reporter in various studies (e.g., Jonuscheit et al., 2003; Wagner et al., 2012), including promoter investigations (Berkner et al., 2007, 2010; Peng et al., 2012; van der Kolk et al., 2020). In contrast to esterase Saci_1116, LacS already exhibits substantial expression levels, both with the N- and C-terminal Twin-Strep tag, in the absence of a 5’-UTR (Supplementary Figure S7). Nevertheless, the addition of 5’-UTRs resulted in a slight increase in LacS production (Supplementary Figure S8). Specifically, the *alba* 5’-UTR led to a 1.6-fold increase in LacS production (Supplementary Figure S8). Remarkably, the effect of the different 5’-UTRs on the production of the two reporter proteins showed a consistent pattern, demonstrating the transferability of the observed 5’-UTR effects to different target proteins. Notably, the degree of increase in protein production was dependent on the target protein.

### 3.5 Application of the *alba* 5’-UTR optimized expression vector for protein production

**Figure 5:**
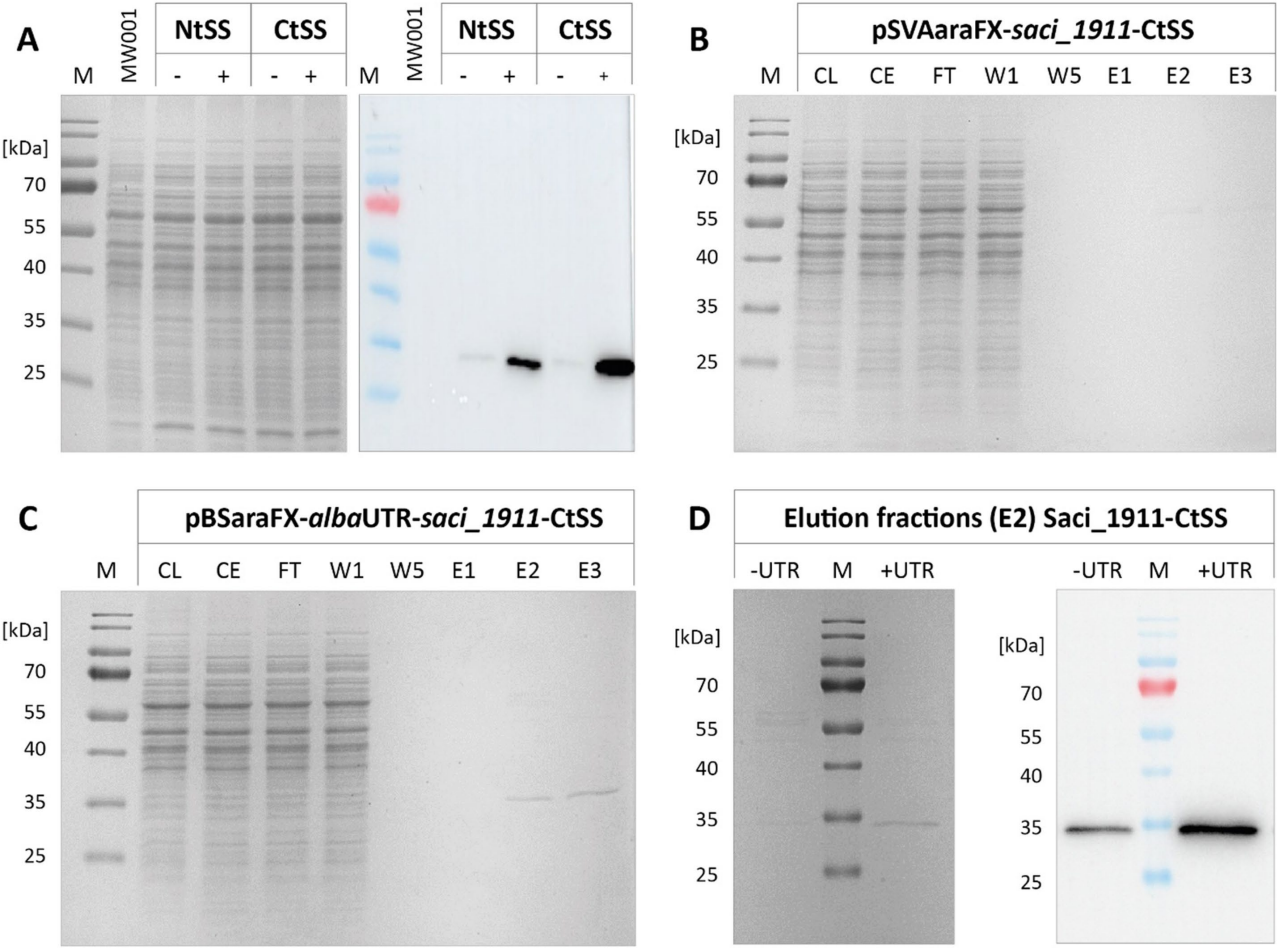
Effect of the *alba* 5’-UTR on the homologous production of glycosyltransferase Saci_1911. **(A)** SDS-PAGE and immunodetection of Twin-Strep-tagged Saci_1911 in cell lysates from *S. acidocaldarius* expressing *saci_1911* without (−) and with *alba* 5’-UTR (+), and with an N-terminal (Nt) or C-terminal (Ct) Twin-Strep tag (SS). *S. acidocaldarius* MW001 without plasmid served as a reference (MW001). **(B,C)** SDS-PAGE gels of Saci_1911-CtSS affinity chromatography fractions after plasmid-based expression in *S. acidocaldarius* without **(B)** and with *alba* 5’-UTR **(C)**. **(D)** SDS-PAGE and immunodetection of Saci_1911-CtSS in affinity chromatography elution fractions (E2). Expression cultures were harvested at OD_600nm_ values of 0.8. Protein was purified from crude extracts using the Strep-Tactin^®^XT 4Flow® resin following the manufacturer’s protocol. M: marker; CL: cell lysate; CE: crude extract; FT: flow-through; W: wash fraction; E: elution fraction, 20 μL each. Theoretical molecular weight of Saci_1911-CtSS: 33.8 kDa.

**Figure 6:**
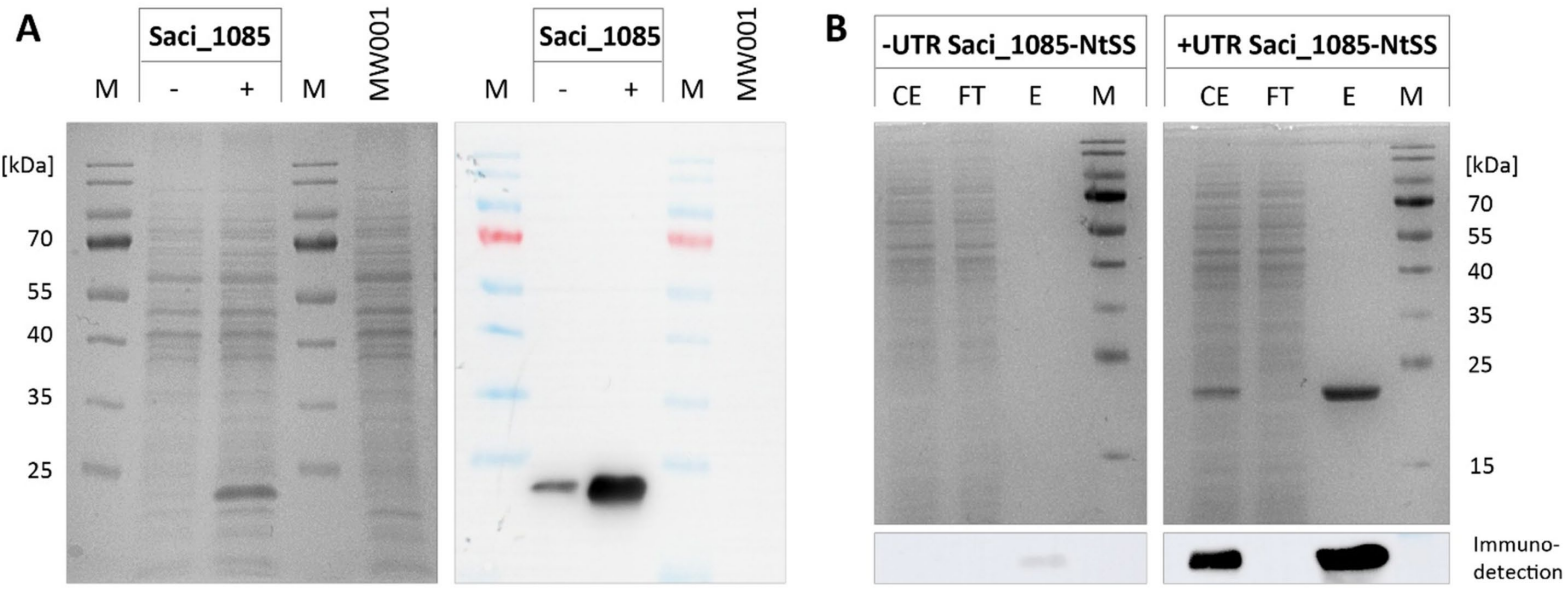
Effect of the *alba* 5’-UTR on the production of hydroxyacyl-CoA dehydratase Saci_1085-NtSS. **(A)** SDS-PAGE and immunodetection of Saci_1085-NtSS in cell lysates from *S. acidocaldarius* expressing *saci_1085* without (−) and with *alba* 5’-UTR (+) and with an N-terminal Twin-Strep tag (NtSS). *S. acidocaldarius* MW001 without plasmid served as a reference (MW001). **(B)** SDS-PAGE of Saci_1085-NtSS affinity chromatography fractions. Protein was purified using Strep-Tactin®XT 4Flow® resin. The relevant image section of Saci_1085-NtSS immunodetection using Strep-Tactin-HRP conjugate is presented below. M: marker; CE: crude extract; FT: flow-through; E: pooled elution fractions; 2 μg protein for +UTR. Theoretical molecular weight of Saci_1085-NtSS: 22.5 kDa.

Our previous experiments illustrated the impact of 5’-UTR-assisted protein production for two different target proteins. To further investigate the effectiveness of the newly modified expression vectors, we screened their impact for nine additional proteins. In several studies, the production of glycosyltransferases (GTs) has been reported as demanding (e.g., Breton et al., 2006; Cobucci-Ponzano et al., 2011a, 2011b; Mestrom et al., 2019). Accordingly, also GTs originating from *S. acidocaldarius* have proven difficult to express in both *E. coli* (Jachlewski, 2013) and *S. acidocaldarius* when using vectors lacking a 5’-UTR (this study). Thus, we utilized the new *alba* 5’-UTR modified expression vectors, designated as pBSaraFX-*alba*UTR-NtSS and -CtSS (Supplementary Figures S9, S10), for GT synthesis. We assessed the production of six GTs from *S. acidocaldarius*, both in absence and in presence of the *alba* 5’-UTR, respectively. For all GTs tested (Saci_1904, Saci_1909, Saci_1911, Saci_1915, Saci_1916, Saci_1921), the presence of *alba* 5’-UTR increased protein amounts significantly, as confirmed by immunodetection of the Twin-Strep-tagged target proteins in cell lysates (Figure 5, Supplementary Figure S11). As a proof of concept, the production and purification of GT Saci_1911 was analysed in more detail. In the case of this GT, its production was higher with a C-terminal compared to an N-terminal tag (Figure 5A). C-terminal tagged Saci_1911 was purified from crude extracts by affinity chromatography, and the different fractions were visualized by SDS-PAGE (Figures 5B,C). Notably, the amount of purified Saci_1911-CtSS increased when cells contained the *alba* 5’-UTR expression plasmid compared to expression in the absence of a 5’-UTR (Figure 5D). The *alba* 5’-UTR-modified expression system was further used for the synthesis of three more proteins, namely glycerol kinase Saci_2033 (Schmerling et al., 2024a), malto-oligosyltrehalose trehalohydrolase (TreZ) from *S. solfataricus* (SSO2093) (Kobayashi et al., 1996; Fang et al., 2006), and hydroxyacyl-CoA dehydratase Saci_1085 (Schmerling et al., 2024b). Similar to the results observed for LacS, the expression of Saci_2033 remained high in both the absence and presence of the *alba* 5’-UTR (Supplementary Figure S12A). TreZ production was clearly enhanced in the presence of *alba* 5’-UTR (Supplementary Figure S12B). In particular, the expression of the hydroxyacyl-CoA dehydratase Saci_1085 was largely improved by the use of the *alba* 5’-UTR-modified expression plasmid, as demonstrated by protein visualization in cell lysates (Figure 6A) and affinity chromatography fractions (Figure 6B). Notably, the synthesis of active Saci_1085 was unsuccessful in *E. coli* (pET15b, N-terminal His_6_-tag), whereas the homogenously expressed enzyme was soluble and active as reversible 3-hydroxyacyl-CoA dehydratase (Schmerling et al., 2024b).

In summary, we observed a noticeable positive impact of the *alba* 5’-UTR on protein expression in nine out of eleven cases. For the remaining two proteins, LacS and Saci_2033, production levels were already high without the 5’-UTR and remained high in its presence. Altogether, these results strongly demonstrate the efficacy of the 5’-UTR modified expression vector and highlight its potential for enhancing protein production across various targets.

### 3.6 Impact of Shine-Dalgarno (SD) motif on protein production

**Figure 7:**
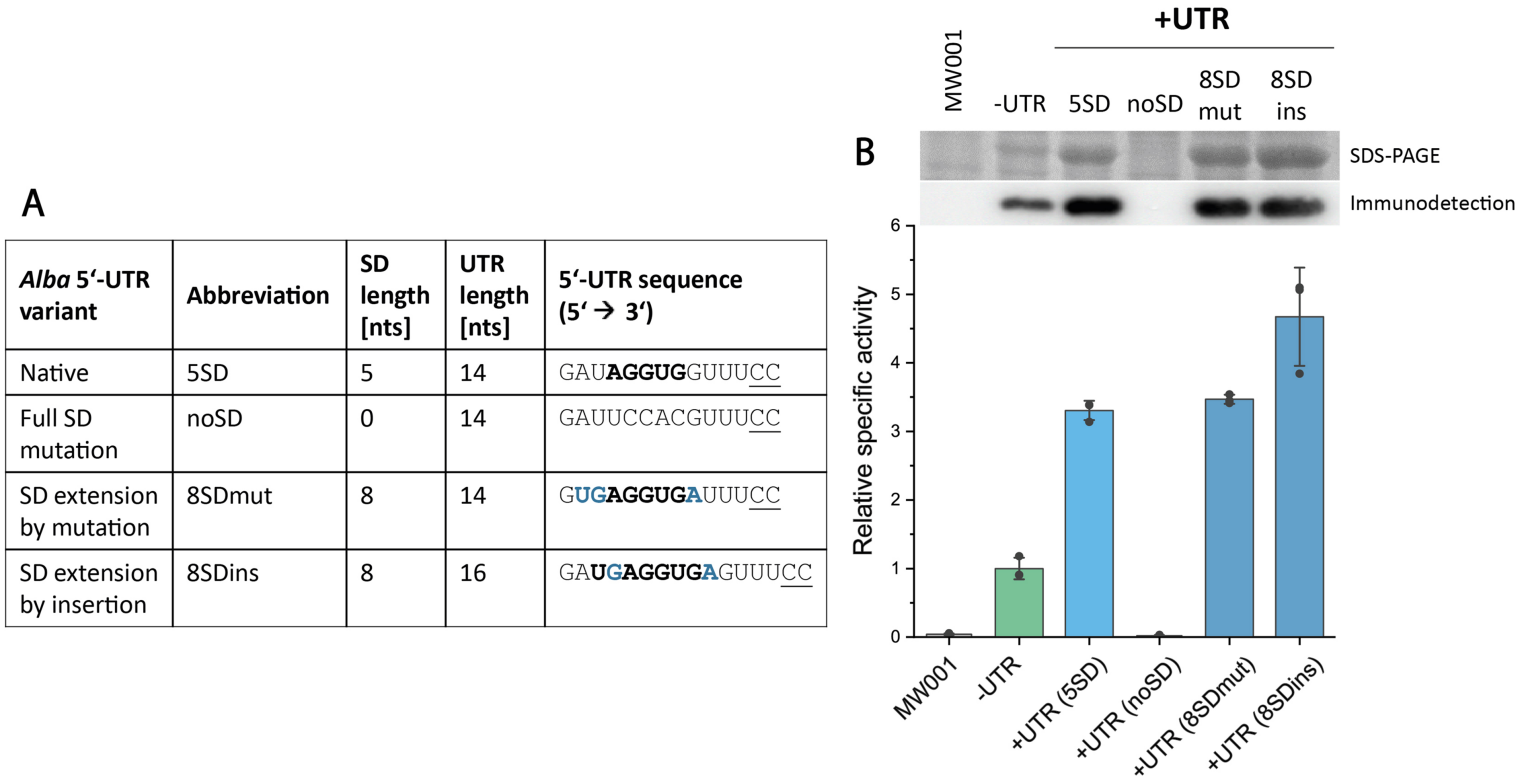
Effect of SD motif sequence alterations within the *alba* 5’-UTR on esterase production. **(A)** Overview of tested *alba* 5’-UTR-SD variants, including SD lengths, UTR lengths, and 5’-UTR nucleotide sequences. **(B)** Esterase activity in cell lysate samples from *S. acidocaldarius* MW001 expression cultures was determined using the continuous *p*NPA assay. Error bars indicate the standard deviation of three biological replicates (*n* = 3), shown as data points. Sections of the respective SDS-PAGE analysis of cell lysates (upper row) and Saci_1116-CtSS immunodetection (lower row) are shown (uncropped images in Supplementary Figure S13). Cultures were harvested at OD_600nm_ values of 0.6–0.8 (log phase). *S. acidocaldarius* MW001 without plasmid served as a reference (MW001).

As previously noted, the native *alba* 5’-UTR contains a 5 nt long SD motif. This prompted us to investigate the relevance of this motif for efficient protein production. Considering the aSD sequence found in *S. acidocaldarius* 16S rRNA, the SD motif “5’ UGAGGUGA 3′” would perfectly complement the aSD over a length of 8 nucleotides (Ma et al., 2002; Schmitt et al., 2020; Wen et al., 2021). To explore the potential enhancement of protein production by extending the SD motif, we constructed *alba* 5’-UTR variants containing 8-nucleotide long SD motifs. The first variant, designated “8SDmut” was generated by site-directed mutagenesis, altering nucleotides neighboring the native 5 nt long SD motif while maintaining the overall UTR length (Figure 7A). The second variant, named “8SDins,” was generated by the insertion of two nucleotides to create an 8-nucleotide long SD motif, thereby increasing the total length of the 5’-UTR. Additionally, the native 5 nt long SD motif was completely mutated to generate a 5’-UTR lacking the SD motif (“noSD” variant) (Figure 7A). Throughout these modifications, the overall GC content of the 5’-UTR was kept constant at 50%. Esterase Saci_1116-CtSS was utilized as reporter protein and its production was assessed by enzymatic activity, SDS-PAGE, and immunodetection (Figure 7B).

Strikingly, the absence of the SD motif within the *alba* 5’-UTR resulted in a complete cessation of protein production, as confirmed by enzymatic measurements and protein visualization (Figure 7, Supplementary Figure S13). One of the 8-nucleotide long SD variants, “8SDins,” facilitated a further increase in protein production compared to the native *alba* 5’-UTR with a 5-nucleotide long SD motif. In contrast, the variant “8SDmut,” generated through nucleotide mutagenesis, displayed protein production levels comparable to those of the native *alba* 5’-UTR (Figure 7B). These results suggest that the sole extension of the SD motif does not account for the observed increase in protein production, indicating the presence of complex effects at the sequence or potentially transcript level. Importantly, our findings underscore the crucial role of the SD motif within the *alba* 5’-UTR in allowing protein synthesis.

## 4 Discussion

In this study, we examined the impact of 5’-UTR sequence insertions into *S. acidocaldarius* P_ara_ expression plasmids on the production of different (reporter) proteins. Our findings elucidate that the integration of SD motif-containing 5’-UTRs from proteins with high cellular abundance significantly enhanced plasmid-based protein synthesis for the majority of tested target proteins.

The utilization of 5’-UTRs and RBS, i.e., preserved Shine-Dalgarno sequences, is prevalent in bacterial expression vectors, such as pBAD, pET, pMAL, pQE, and pGEX plasmids for heterologous expression in *E. coli,* or pHT and pBSMul1 expression plasmids in *Bacillus subtilis* (Brockmeier et al., 2006; Yang et al., 2021). Consequently, the influence of bacterial 5’-UTR sequence elements, i.e., SD motifs or spacer sequences, has been extensively investigated in several bacterial organisms over the last decades (e.g., Wu and Janssen, 1997; Sakai et al., 2001; Khosa et al., 2018; Volkenborn et al., 2020). In contrast, 5’-UTRs have been less considered and studied as a relevant feature for gene overexpression in Archaea. However, this disparity is likely related to the fact that, unlike bacteria, many common archaeal model organisms, including Haloarchaea, Thermoproteales, and Sulfolobales, primarily generate leaderless gene transcripts (e.g., 72% for *H. volcanii* (Schmitt et al., 2020), or 69% for *S. solfataricus* (Wurtzel et al., 2010); Slupska et al., 2001; Brenneis et al., 2007; Kramer et al., 2014). Consequently, 5’-UTRs were considered less relevant for protein synthesis and as a feature of archaeal expression vectors. However, some existing archaeal expression vectors do generate leadered mRNA transcripts for gene overexpression, facilitated by the presence of an extended “promoter” sequence that leads to the generation of a 5’-UTR. For instance, the sequence of the heat-inducible tf55 promoter in *S. solfataricus* expression vectors generates an 18 nt long 5’-UTR harbouring a putative 4 nt long SD motif (Jonuscheit et al., 2003). The existing sugar-inducible *S. acidocaldarius* expression vectors featuring P_ara_ from *saci_2122* and P_mal_ (maltose) from *saci_1165* generate leaderless transcripts, as determined by available RNA sequencing data of corresponding gene transcripts from the genome (Cohen et al., 2016). However, in the case of P_xyl_ (promoter from *saci_1938*) (van der Kolk et al., 2020), a leadered transcript devoid of an SD motif is generated (34 nts, Cohen et al., 2016).

Here, we introduced SD motif-containing 5’-UTR sequences from genes encoding highly abundant proteins into the P_ara_ *S. acidocaldarius* expression vector. All five tested 5’-UTR insertions demonstrated enhanced production of esterase Saci_1116, showing a two- to four-fold increase. Notably, the use of *alba* (*saci_1322*) 5’-UTR, encoding the most abundant protein in *S. acidocaldarius* according to full proteome data (Schmerling et al., 2024a), resulted in a four-fold increase in the yield of soluble, active esterase. In agreement, in *Methanococcus maripaludis* the replacement of a wild-type 5’-UTR by 5’-UTRs from highly expressed genes (e.g., *slp* (S-layer protein) and *hmmA* (histone A)) led to increased protein production (Akinyemi et al., 2021). Thus, both in *M. maripaludis* and in *S. acidocaldarius* the utilization of 5’-UTRs originating from highly expressed genes resulted in higher plasmid-based target protein production. 

In contrast, tested native 5’-UTRs from *H. volcanii* (*hlr* (hoxA-like transcriptional regulator) and *hp* (conserved hypothetical protein)) without an SD motif reduced reporter protein amounts by approximately two-fold when compared to leaderless transcripts (Brenneis and Soppa, 2009). However, two out of four artificial 5’-UTR sequences (20 nts, random sequence, no SD) increased reporter activity by factors of two and five, respectively, in *H. volcanii* (Brenneis et al., 2007; Hering et al., 2009). Furthermore, 5’-UTR sequences from genes involved in gas vesicle formation in *Halobacterium salinarum* and a short 5′-terminal nucleotide sequence (4 nts) from heat shock protein 70 (*hsp70*) of *Natrinema* sp. J7 decreased reporter production in *H. volcanii* (Born and Pfeifer, 2019; Chen et al., 2015) (compare Supplementary Tables S6, S7 for literature review). Notably, modifications to the nucleotide identities at positions “-1” and “-2” within the short *hsp70* 5’-UTR revealed varied effects on protein production in *H. volcanii* (Chen et al., 2015), hence providing a more complex picture.

In *S. islandicus*, mutating the two nucleotides upstream of the translation start codon (from “AU” to “CC”) within the 6 nt long 5’-UTR of P_araS_ (Peng et al., 2009) reduced protein levels by approximately 45%, attributed to a 50% decrease in transcript levels but a 30% increase in translational efficiency (Ao et al., 2013). Notably, 5’-UTRs were previously discussed to increase the relative promoter strength of the *araS* promoter in *S. solfataricus* by acting as initiator element that can contribute to promoter strength (Peng et al., 2009; Ao et al., 2013). In our study, we also made changes to the two nucleotides immediately upstream of the translation start codon within the *alba* 5’-UTR to retain the *Nco*I cloning site. However, we did not observe a significant effect of this modification on esterase production. Thus, the impact of the nucleotide identities directly upstream of the translation start codon is likely unique to the overall 5’-UTR sequence and its effects on different regulatory levels, such as transcription initiation, transcript stability or translation initiation, rendering it rather complex to predict. Further investigations, including the determination of transcript levels and stabilities, will help to shed more light into these aspects in the future.

Occasionally, the relevance of the SD (RBS) sequence on protein production has been investigated for some 5’-UTRs in Archaea (Supplementary Table S7). Concerning Sulfolobales, previous studies have investigated SD mutations or insertions in *S. solfataricus* and *S. islandicus*, respectively. For *S. solfataricus*, the substitution of two nucleotides of the SD motifs of ORF104 or ORF143 of a bicistronic mRNA (8 and 7 nt long SD motifs, respectively) entirely abolished their translation in a cell-free *in vitro* translational system (Condó et al., 1999). Consistent with this finding, we observed no protein production upon complete mutagenesis of the SD motif within the *alba* 5’-UTR in *S. acidocaldarius*. The insertion of 14 nts, including an 8 nt long SD motif, into an existing 6 nt long 5’-UTR sequence in P_araS_ expression vectors led to a three-fold increase in reporter activity (*β*-galactosidase) in *S. islandicus* (Peng et al., 2012; Peng et al., 2017). Similarly, we found that the insertion of two additional nucleotides to the *alba* 5’-UTR, thereby extending the length of the SD motif and the overall UTR length, increased the yield of esterase. In *Thermococcus kodakarensis*, mutations to the second nucleotide of a 6-nucleotide SD motif significantly decreased protein production (Santangelo et al., 2008). However, in *H. volcanii*, effects of SD motif modifications in different 5’-UTR sequences were more ambiguous (Kramer et al., 2014; Sartorius-Neef and Pfeifer, 2004) (compare Supplementary Table S7). Concluding, the relevance of SD motifs within archaeal 5’-UTRs remains uncertain, emphasizing the need to further investigate their impact on protein expression at the different regulatory levels.

Altogether, reports on the influence of 5’-UTRs in Archaea suggest that, compared to leaderless transcripts, 5’-UTRs do not consistently enhance protein production. Multiple factors may contribute to the differential effects of 5’-UTR sequences on protein production efficiency, including complex regulatory mechanisms at the posttranscriptional level (e.g., secondary structures, transcript stability), as well as alterations to the ribosome docking site for translation initiation. This complexity likely applies to *S. acidocaldarius* as well; however, our preselection of 5’-UTR sequences from genes with high protein abundance probably precluded the appearance of 5’-UTRs that might reduce protein production.

## 5 Conclusion

For many archaeal genes, heterologous expression in mesophilic bacterial strains, such as *E. coli*, is effective, with established straightforward purification protocols, including heat precipitation for thermophilic proteins. However, there are numerous examples where attempts to express archaeal proteins in mesophilic bacterial hosts fail. Thus, archaeal expression systems are needed for synthesizing demanding archaeal proteins, often with biotechnological potential, that cannot be efficiently produced in bacterial hosts. Our study showcases the optimization of plasmid-based protein production through the incorporation of SD motif-containing 5’-UTR sequences in the archaeal expression host *S. acidocaldarius*. The integration of all tested 5′-terminal sequences into P_ara_ expression vectors led to a significant enhancement in protein yield, exemplified by the substantial increase in the production of esterase Saci_1116. Notably, among the five different 5′-terminal sequences examined, the 5’-UTR of the Alba-encoding gene *saci_1322* was found to be highly efficient and reliable. The *alba* 5’-UTR containing expression vector proved to be effective for the synthesis of different Sulfolobales target proteins. Overall, the application of 5’-UTR-optimized expression vectors holds promise for facilitating the synthesis of other challenging thermostable proteins in the future.

## Data Availability

The original contributions presented in the study are included in the article/Supplementary material, further inquiries can be directed to the corresponding authors. Nucleotide sequences and the vector DNA are available to the scientific community upon request.
