# Supplementary material for: 5′-untranslated region sequences enhance plasmid-based protein production in Sulfolobus acidocaldarius
Source: Front Microbiol. 2024 Nov 25;15:1443342. doi: 10.3389/fmicb.2024.1443342 (PMC11627041; doi:10.3389/fmicb.2024.1443342)
Supplement: Supplementary file 1 [file Data_Sheet_1.PDF]

Supplementary Material

5’-untranslated region sequences enhance plasmid-based protein production in *Sulfolobus acidocaldarius*

Laura Kuschmierz[1*], Alexander Wagner[1], Christian Schmerling[1], Tobias Busche[2], Jörn Kalinowski[2], Christopher Bräsen[1] and Bettina Siebers[1*]

[1]Molecular Enzyme Technology and Biochemistry (MEB), Environmental Microbiology and Biotechnology (EMB), Centre for Water and Environmental Research (CWE), Faculty of Chemistry, University of Duisburg-Essen, Essen, Germany

[2]Microbial Genomics and Biotechnology, Center for Biotechnology (CeBiTec), Bielefeld University, Bielefeld, Germany

***Correspondence:**
Laura Kuschmierz
laura.kuschmierz@uni-due.de

Bettina Siebers
bettina.siebers@uni-due.de

1 Supplementary Data

2 Supplementary Figures and Tables

2.1 Supplementary Figures

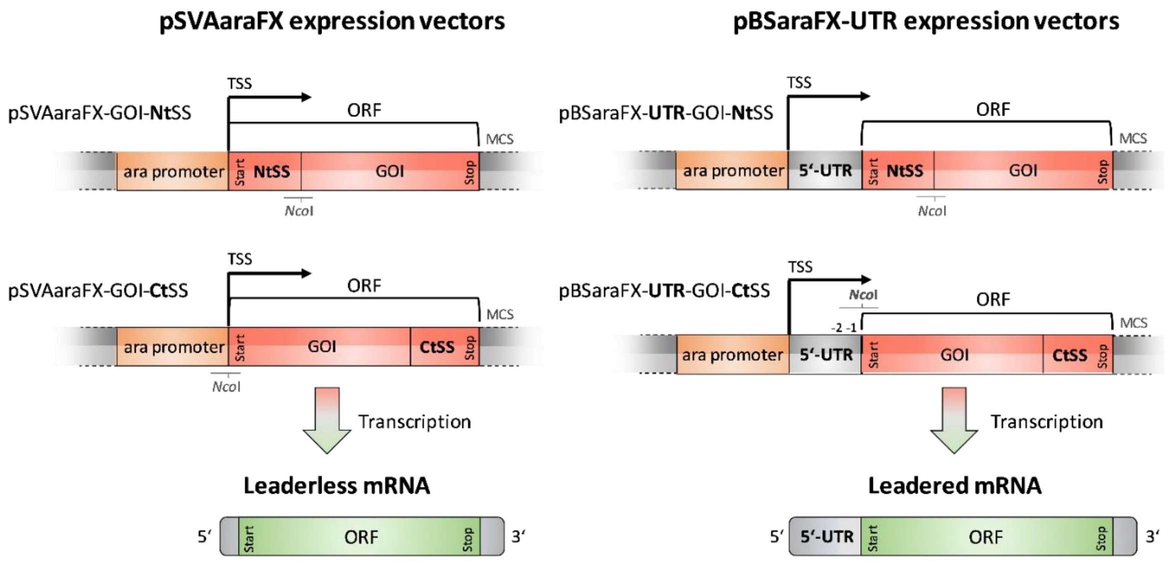

Supplementary Figure 1. Structural elements of pSVAaraFX- and pBSaraFX-UTR expression cassettes and the generation of leaderless or leadered mRNAs, respectively. Upper row: Twin-Strep tag expression vectors with an N-terminal tag location; lower row: C-terminal tag location. The position of the *Nco*I restriction site and -2 and -1 nucleotides (upstream of the translation start codon) within the 5'-UTR of pBSaraFX-UTR-GOI-CtSS are indicated. ara promoter: *saci_2122* promoter; TSS: transcription start site; ORF: open reading frame; NtSS: N-terminal Twin-Strep tag; GOI: gene of interest; CtSS: C-terminal Twin-Strep tag; Start: translation start codon; Stop: translation stop codon; MCS: multiple cloning site.

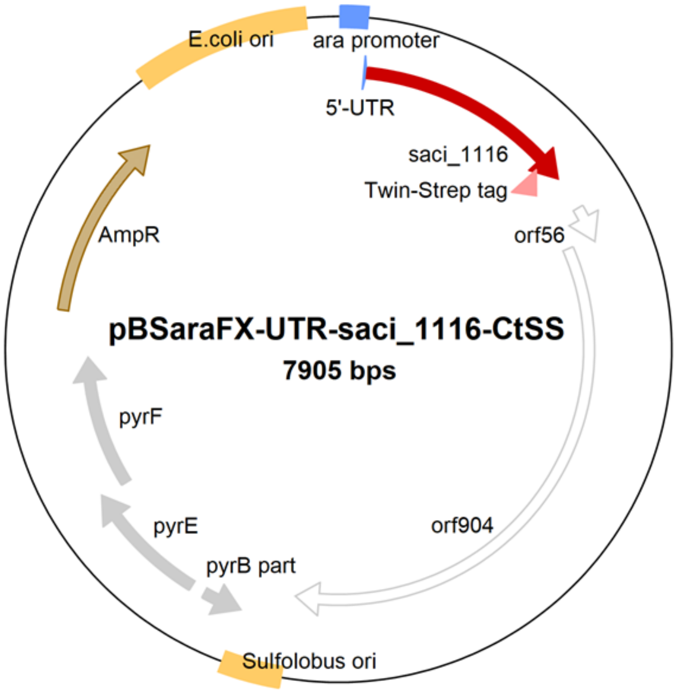

Supplementary Figure 2. Map of pBSaraFX-*alba*UTR-*saci_1116*-CtSS.
The gene of interest, *saci_1116*, was cloned in-frame under control of the pentose-inducible ara promoter (promoter of *saci_2122*). A 5'-UTR sequence, here the *alba* (*saci_1322*) 5'-UTR, was cloned directly upstream of the translation start codon (ATG). The C-terminal end of the GOI (reporter gene *saci_1116*) is followed by a sequence that encodes the C-terminal Twin-Strep tag (-CtSS). The asterisk within the Twin-Strep tag coding element indicates that this sequence location is variable (downstream of the GOI in -CtSS constructs, upstream of the GOI in -NtSS expression constructs) (Fig. S1). Classical shuttle vector elements, such as an origin of replication (ori) for *E. coli* and *S. acidocaldarius*, the ampicillin resistance cassette (AmpR) for selection in *E. coli* as well as *pyrEF* and part of *pyrB* (containing the *pyrEF* promoter sequence) from *Saccharolobus solfataricus* for selection in *S. acidocaldarius* MW001 are indicated. *orf56*, encoding a DNA-binding protein, and *orf904*, encoding a multifunctional replication protein, originate from plasmid pRN1 from *S. islandicus* and regulate plasmid copy number (Lipps, 2004). Clone Manager 7 (Sci Ed Software, USA) was used to generate the figure.

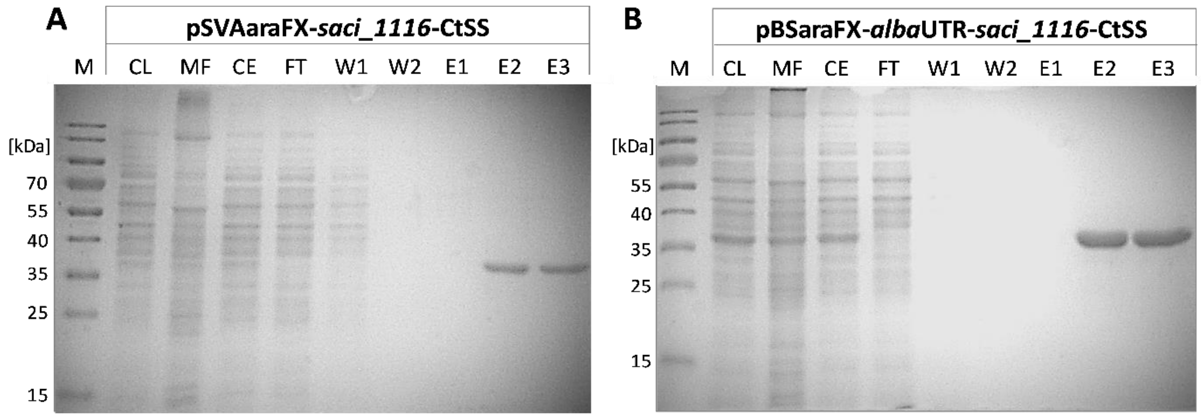

Supplementary Figure 3. Effect of *alba* 5'-UTR on homologous production of the esterase reporter protein Saci_1116-CtSS. Coomassie-stained SDS-PAGE gel of purified Saci_1116-CtSS after expression in *S. acidocaldarius* MW001 without a 5'-UTR; (**A**) and with the *alba* 5'-UTR (**B**). Expression cultures were grown under the same growth conditions in Brock medium (200 mL each, pH 3.0) supplemented with 0.1% (w/v) NZA and 0.3 (w/v) D-xylose at 140 rpm and 76°C and harvested simultaneously at OD_{600nm} values of 1.0 and 0.9, respectively. A theoretical turbidity (OD_{600}) of 5 was applied for cell lysis by sonication. Protein purification from crude extracts (soluble fraction) was performed using the Strep-Tactin®XT Superflow® Kit according to the manufacturer's protocol. (M: marker (protein ladder); CL: cell lysate (whole cell sample after lysis); CE: crude extract (soluble fraction); MF: membrane fraction (pellet after cell lysis); FT: flow-through fraction of affinity chromatography; W: wash fraction; E: elution fraction, 15 µL each). Theoretical MW of Saci_1116-CtSS: 36.8 kDa.

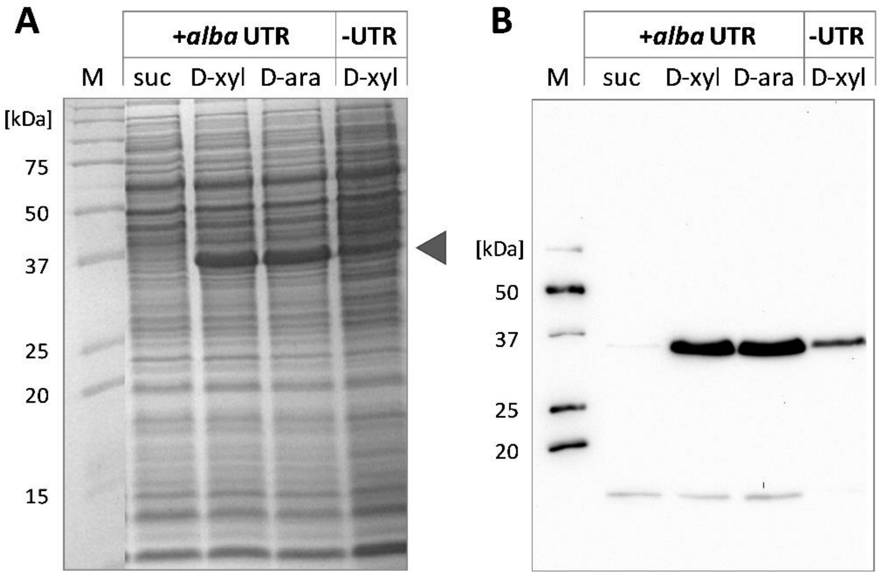

Supplementary Figure 4. Effect of *alba* 5'-UTR insertion on P_{ara} functionality and activity. *S. acidocaldarius* MW001 was transformed with pBSaraFX-*alba*UTR-*saci_1116*-CtSS (+*alba* UTR) or pSVAaraFX-*saci_1116*-CtSS (-UTR). Expression cultures were grown in Brock medium (pH 3.0) with 0.1% (w/v) NZA and different carbon sources (suc: 0.3% (w/v) sucrose; D-xyl: 0.3% (w/v) D-xylose, D-ara: 0.05% (w/v) D-arabinose) at 76°C. **A**) Coomassie-stained SDS-PAGE gel of cell lysate samples. **B**) Immunodetection of the Twin-Strep-tagged esterase Saci_1116 using Strep-Tactin-HRP conjugate. Theoretical MW of Saci_1116-CtSS: 36.8 kDa. M: marker (protein ladder).

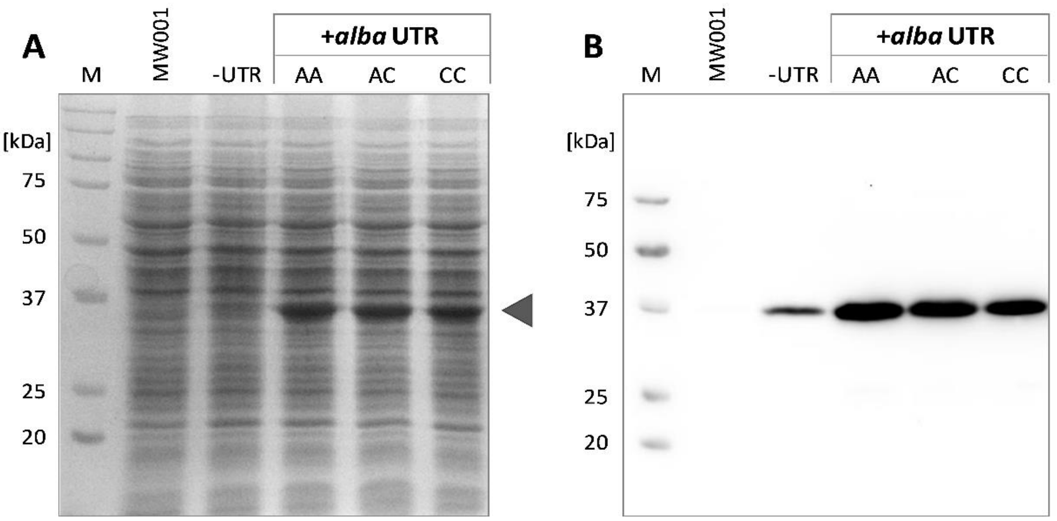

Supplementary Figure 5. Influence of -2 and -1 nucleotide identities in *alba* 5'-UTR on esterase reporter protein production. Expression studies were performed using different *saci_1116*-CtSS expression constructs, either without 5'-UTR (-UTR) or with the *alba* 5'-UTR with different nucleotide sequences at the -2 and -1 positions, i.e. AA, AC, or CC. Saci_1116-CtSS production was visualized by SDS-PAGE and Coomassie staining (**A**), and immunodetection (Strep-Tactin HRP conjugate) (**B**). M: marker (protein ladder).

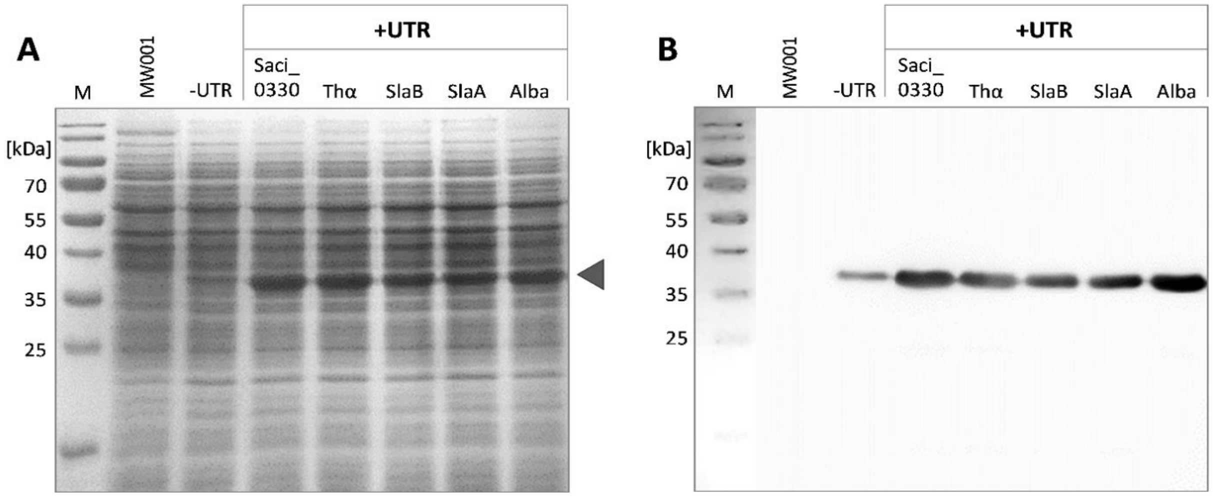

Supplementary Figure 6. Screening of five different 5'-UTR sequences for the production of C-terminal tagged esterase reporter protein. A) Visualization of proteins in cell lysates by SDS-PAGE and Coomassie staining. **B**) Immunodetection of C-terminal tagged esterase in cell lysate samples (Strep-Tactin HRP conjugate). Saci_1116-CtSS production was performed using constructs without (-UTR) or with a 5'-UTR (**+UTR**). 5'-UTR sequences of genes *saci_0330*, *saci_1401* (Thα), *saci_2354* (SlaB), *saci_2355* (SlaA) and *saci_1322* (Alba), respectively, were modified in favor of the *Nco*I site (Fig. 4; Tab. S4) and inserted into the *saci_1116*-CtSS expression plasmid directly upstream of the GOI start codon. M: marker (protein ladder).

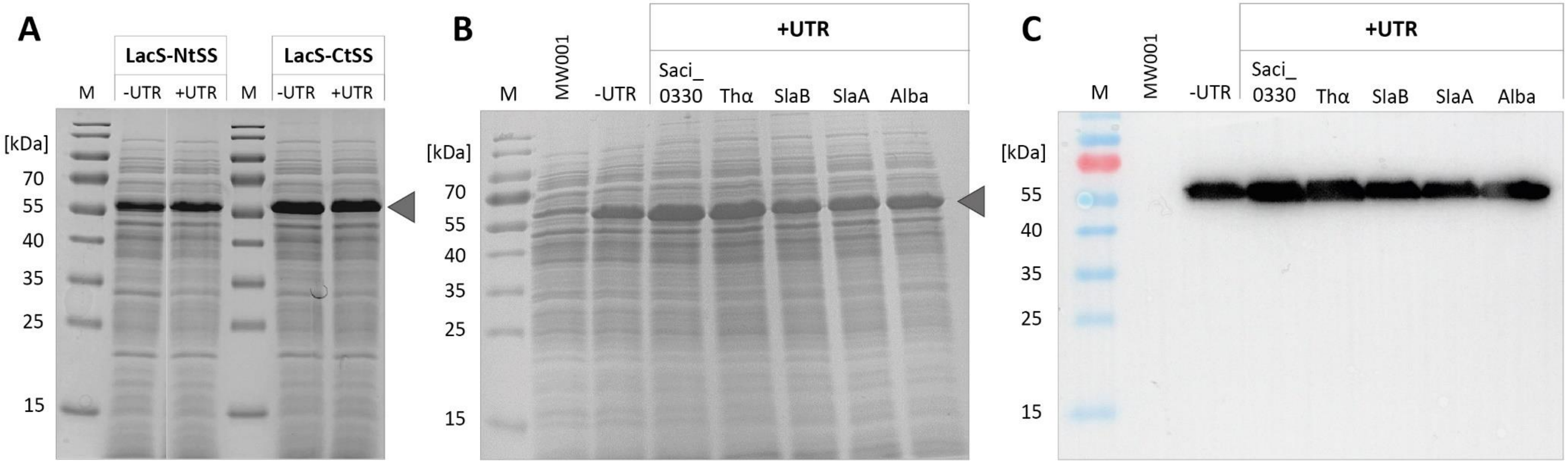

Supplementary Figure 7. Effect of 5'-UTRs on the production of LacS from *Saccharolobus solfataricus*. A) Coomassie-stained SDS-PAGE gel of LacS-SS in cell lysates from *S. acidocaldarius* expressing SSO3019 (*lacS*) without and with *alba* 5'-UTR, and with an N-terminal (Nt) or C-terminal (Ct) Twin-Strep tag (SS). **B, C**) Screening of five different 5'-UTR sequences for the production of LacS-CtSS. Analysis was performed using constructs without (-UTR) or with a 5'-UTR (+UTR). The 5'-UTR sequences from *saci_0330*, *saci_1401* (Thα), *saci_2354* (SlaB), *saci_2355* (SlaA), and *saci_1322* (Alba) were inserted upstream of the *lacS*-CtSS start codon in the expression plasmid. Proteins in cell lysates were visualized by SDS-PAGE (B) and immunodetection using Strep-Tactin HRP conjugate (C). *S. acidocaldarius* MW001 without plasmid served as a reference (MW001). Theoretical molecular weight: LacS-SS: 60 kDa. M: marker (protein ladder).

A

5'-UTR	Inserted 5'-UTR sequence (5' → 3')	UTR length [nts]	SD length [nts]	Spacer length [nts]	Spacer GC content [%]	UTR GC content [%]
Saci_0330	AAA**AGGUGA**CCCAACC	16	6	8	63	50
Thα	AGAAAC**GGUG**UCUCCC	16	4	6	67	56
SlaB	AAUUUAUAACAUAUCG**GGUG** UAUGUCC	27	4	7	43	33
SlaA	GAAA**GUGA**AAACC	13	4	6	33	38
Alba	GAU**AGGUG**GUUUCC	14	5	6	50	50

B

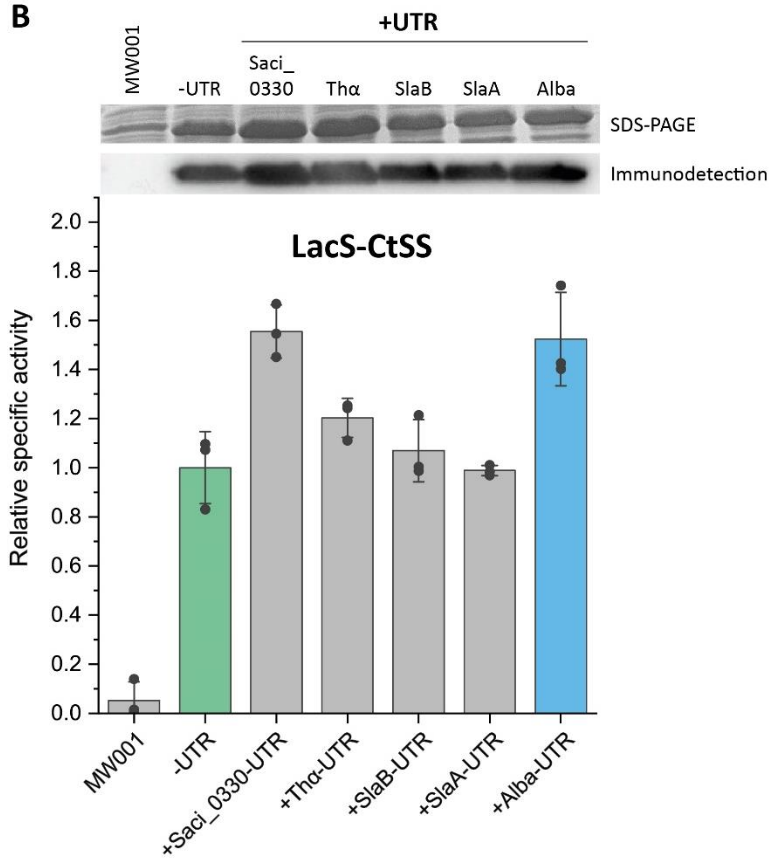

Supplementary Figure 8. Screening of 5'-UTR sequences for the production of C-terminal tagged LacS from *Saccharolobus solfataricus*. A) Structural properties of the tested 5'-UTR sequences, including gene IDs and nucleotide changes at positions -2 and -1 (to "CC", underlined), presence of Shine-Dalgarno (SD) motifs (bold), UTR lengths, putative SD motif lengths, spacer lengths, and GC contents of spacers and total UTR sequences. **B**) Analysis of LacS-CtSS production using constructs without (-UTR) or with a 5'-UTR (+UTR). The 5'-UTR sequences from *saci_0330*, *saci_1401* (Thα), *saci_2354* (SlaB), *saci_2355* (SlaA), and *saci_1322* (Alba) were inserted upstream of the *lacS*-CtSS start codon in the expression plasmid. β-Galactosidase activity was determined in crude extracts with *p*NPG as substrate. The activity without a 5'-UTR was set to "1" (1.4 U/mg), and other activities were normalized accordingly. Error bars represent the standard deviation from three biological replicates (*n*=3), shown as data points. Relevant sections of the SDS-PAGE analysis (upper row) and immunodetection (lower row) are shown (uncropped images in Fig. S7).

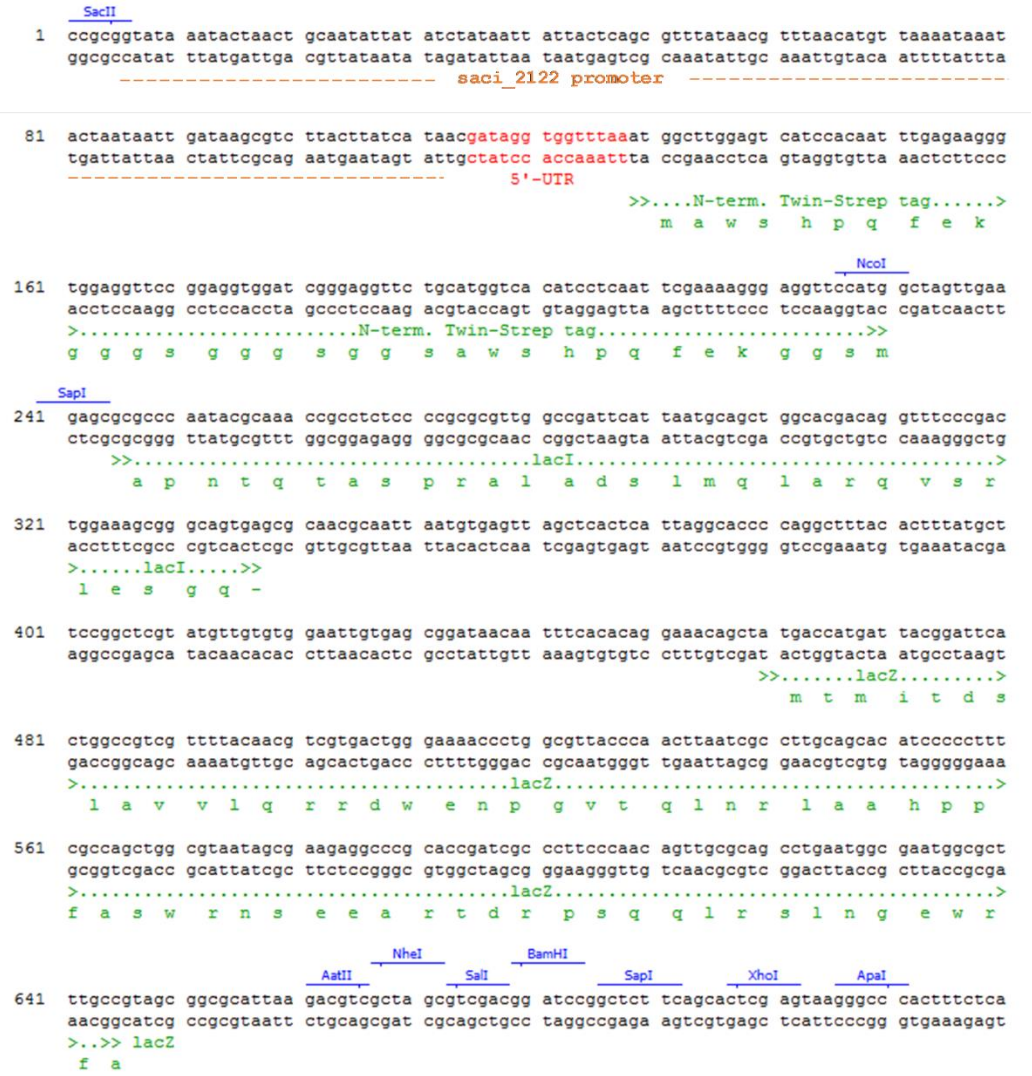

Supplementary Figure 9. Important features of the pBSaraFX-*alba*UTR-NtSS expression vector. The following sequence features are indicated: the *saci_2122* promoter sequence, the inserted 5'-UTR sequence from *alba* (*saci_1322*), the nucleotide as well as derived amino acid sequence of the N-terminal located Twin-Strep tag, the *lacI* and *lacZ* fragments enabling blue-white screening in *E. coli* and the downstream multiple cloning site. Restriction sites are displayed. Clone Manager 7 (Sci Ed Software, USA) was used to generate the figure.

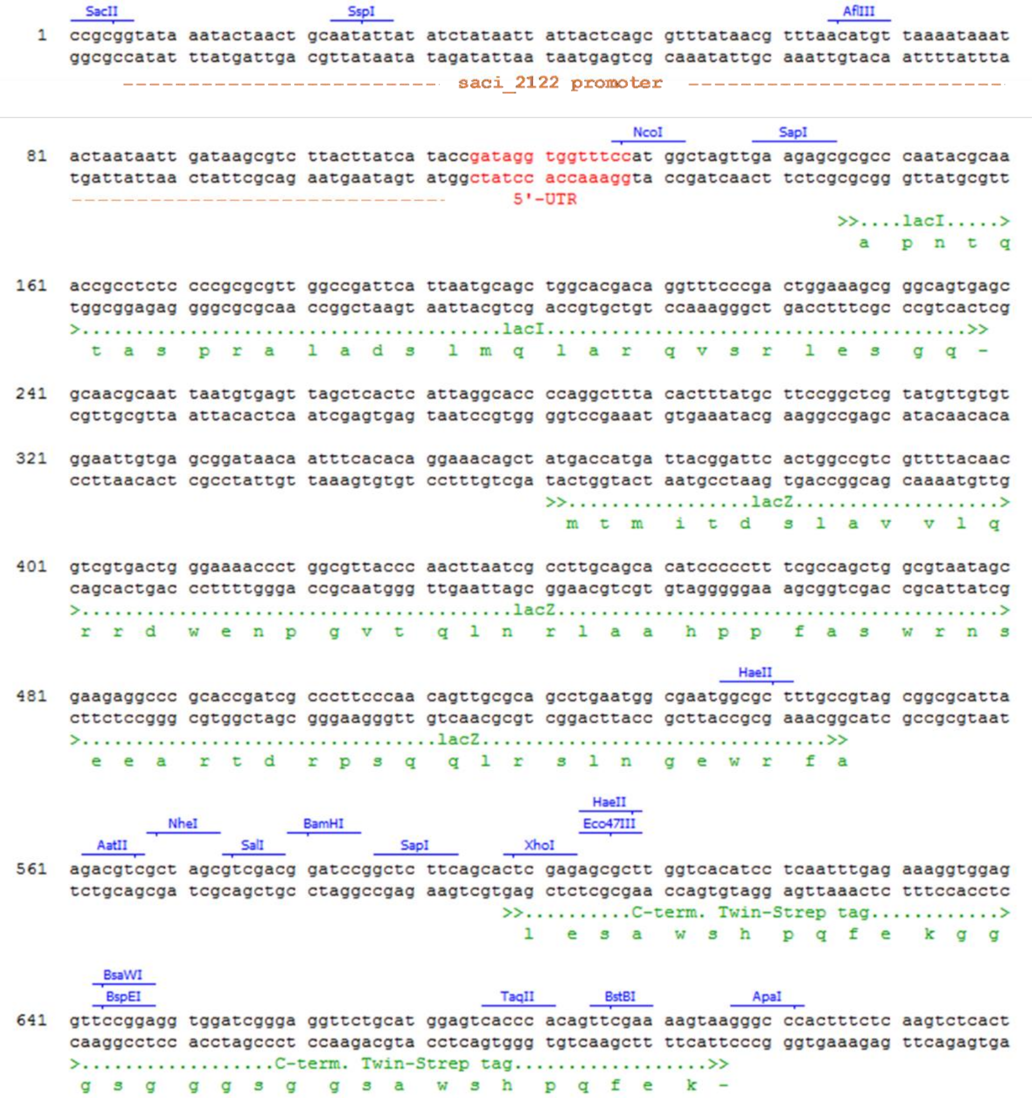

Supplementary Figure 10. Important features of the pBSaraFX-*alba*UTR-Ct SS expression vector. The following sequence features are indicated: the *saci_2122* promoter sequence, the inserted and modified (-2 and -1 nucleotides) 5'-UTR sequence from *alba* (*saci_1322*), the *lacI* and *lacZ* fragments enabling blue-white screening in *E. coli*, the nucleotide as well as the amino acid sequence of the C-terminal located Twin-Strep tag and the downstream multiple cloning site. Restriction sites are displayed. Clone Manager 7 (Sci Ed Software, USA) was used to generate the figure.

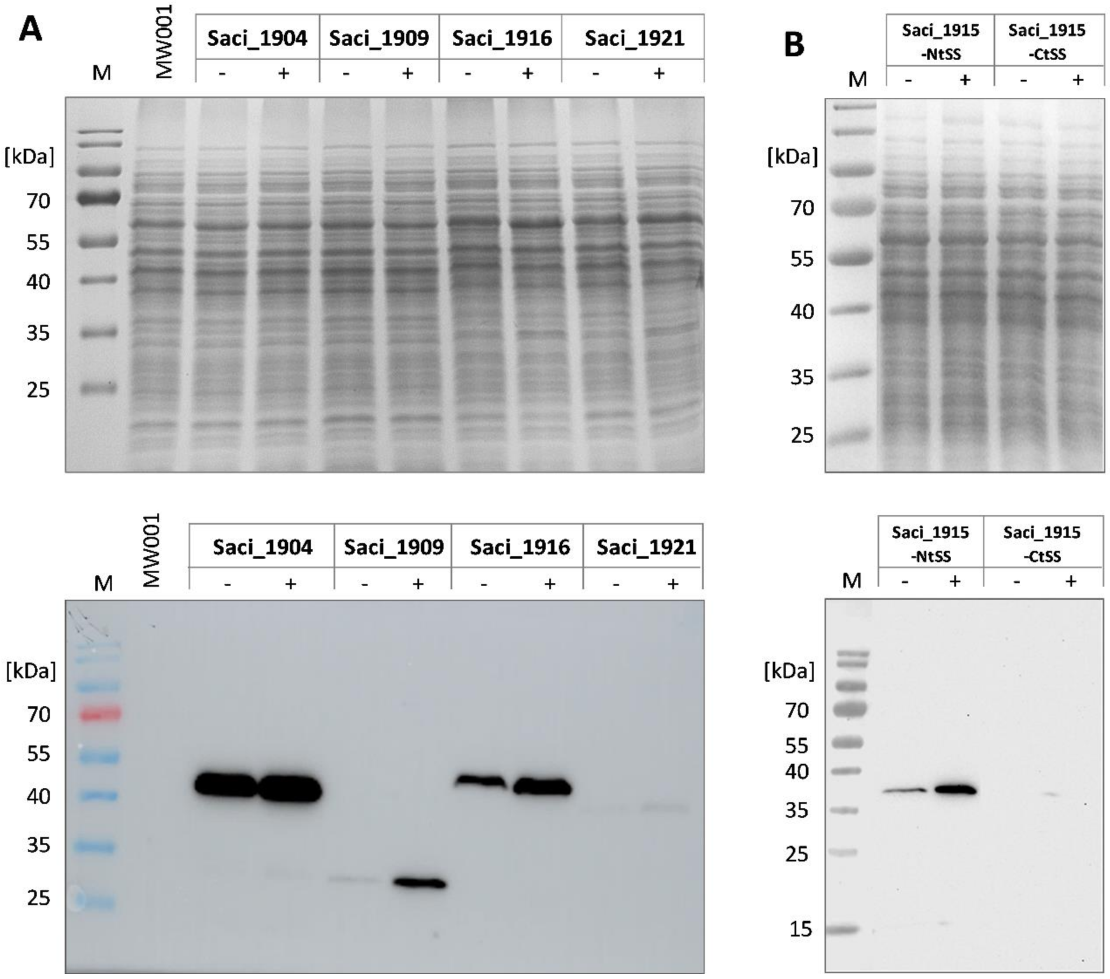

Supplementary Figure 11. Effect of the *alba* 5'-UTR on the homologous production of glycosyltransferases. A) SDS-PAGE and immunodetection of Saci_1904-CtSS, Saci_1909-NtSS, Saci_1916-NtSS, and Saci_1921-NtSS in cell lysates from *S. acidocaldarius* cultures without (-) and with *alba* 5'-UTR (+) in expression plasmids. *S. acidocaldarius* MW001 without plasmid served as a reference (MW001). All cell samples were normalized to an OD_{600nm} value of 10, and 15 µL of each sample were applied to SDS-PAGE. **B)** SDS-PAGE and immunodetection of Saci_1915-SS in cell lysates from *S. acidocaldarius* expressing *saci_1915* without (-) and with *alba* 5'-UTR (+), and with either an N-terminal (Nt) or C-terminal (Ct) Twin-Strep tag (SS). Theoretical molecular weights: Saci_1904-CtSS: 46.2 kDa, Saci_1909-NtSS: 31.6 kDa, Saci_1916-NtSS: 54.6 kDa, Saci_1921-NtSS: 47.7 kDa, Saci_1915-SS: 40.7 kDa. M: marker (protein ladder).

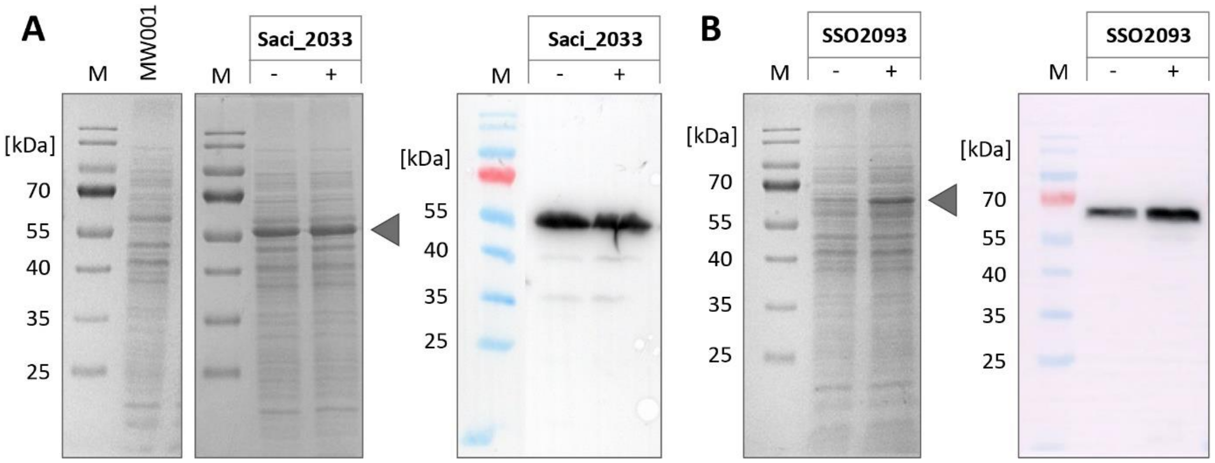

Supplementary Figure 12. Effect of the *alba* 5'-UTR on the production of glycerol kinase Saci_2033 and malto-oligosyltrehalose trehalohydrolase TreZ from *Saccharolobus solfataricus*. A) SDS-PAGE and immunodetection of Saci_2033-CtSS in cell lysates from *S. acidocaldarius* expression cultures without (-) and with *alba* 5'-UTR (+). *S. acidocaldarius* MW001 without plasmid was used as a reference (MW001). All cell samples were normalized to an OD_{600nm} value of 10, and 15 µL of each sample were loaded onto the SDS-PAGE. **B)** SDS-PAGE and immunodetection of TreZ (SSO2093-NtSS) in cell lysates from *S. acidocaldarius* expressing SSO2093 without (-) and with *alba* 5'-UTR (+), and with an N-terminal Twin-Strep tag. Theoretical molecular weights: Saci_2033-CtSS: 58.6 kDa, SSO2093-NtSS: 67.9 kDa. M: marker (prestained protein ladder).

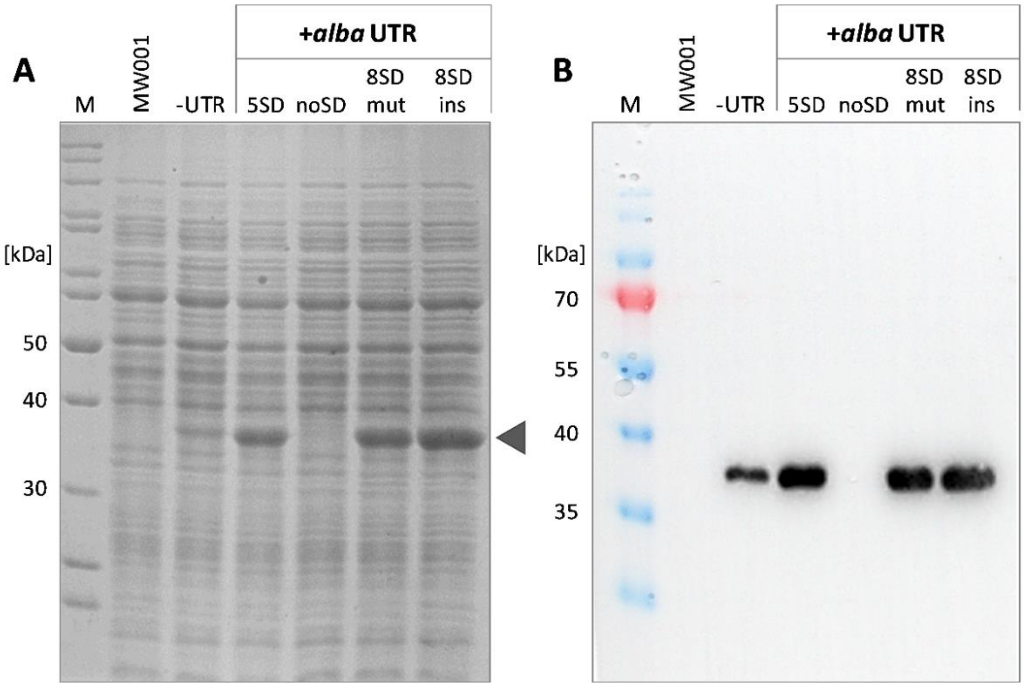

Supplementary Figure 13. **Influence of SD motif variation within *alba* 5’-UTR on esterase production. A**) Protein visualization in cell lysates by SDS-PAGE and Coomassie staining. **B**) Immunodetection of C-terminal tagged esterase in cell lysate samples (Strep-Tactin HRP conjugate). Saci_1116-CtSS production was conducted using constructs without (-UTR) or with different variants of *alba* 5’-UTR (**+*alba* UTR**), with modifications to the 5’-UTR sequence regarding the SD motif (Fig. 7, main text). M: marker (protein ladder).

2.2 Supplementary Tables

Supplementary Table 1. Plasmids used in this study.

Name	Description
pSVAaraFX-CtSS	Cloning vector without 5'-UTR sequence, with *saci_2122* promoter, C-terminal Twin-Strep tag, and *lacI*-*lacZ* from *E. coli* for blue-white screenings (van der Kolk et al., 2020)
pSVAaraFX-NtSS	Cloning vector without 5'-UTR sequence, with *saci_2122* promoter, N-terminal Twin-Strep tag, and *lacI*-*lacZ* from *E. coli* for blue-white screenings (van der Kolk et al., 2020)
pSVAaraFX-*saci_1116*-CtSS	*saci_1116* expression plasmid based on pSVAaraFX-CtSS; silent mutation at nucleotide position 822 in *saci_1116*: T → C, deletion of *Nco*I site
pSVAaraFX-*saci_1116*-NtSS	*saci_1116* expression plasmid based on pSVAaraFX-NtSS; silent mutation at nucleotide position 822 in *saci_1116*: T → C, deletion of *Nco*I site
pBSaraFX-*alba*UTR-CtSS	Cloning vector pBSaraFX-CtSS with 5'-UTR from *saci_1322* (Alba), -2 and -1 nucleotides: CC; with *saci_2122* promoter, C-terminal Twin-Strep tag and *lacI*-*lacZ* from *E. coli* for blue-white screenings
pBSaraFX-*alba*UTR-NtSS	Cloning vector pBSaraFX-NtSS with 5'-UTR from *saci_1322* (Alba), -2 and -1 nucleotides: AA, with *saci_2122* promoter, N-terminal Twin-Strep tag and *lacI*-*lacZ* from *E. coli* for blue-white screenings
pBSaraFX-*alba*UTR-*saci_1116*-CtSS	pSVAaraFX-*saci_1116*-CtSS with 5'-UTR insertion from *saci_1322* (14 nt; Alba), -2 and -1 nucleotides modified for cloning (*Nco*I site): AA → CC
pBSaraFX-*alba*UTR-*saci_1116*-NtSS	pSVAaraFX-*saci_1116*-NtSS with 5'-UTR insertion from *saci_1322* (Alba), -2 and -1 nucleotides: AA
pBSaraFX-*saci_0330*UTR-*saci_1116*-CtSS	pSVAaraFX-*saci_1116*-CtSS with 5'-UTR insertion from *saci_0330* (16 nt), -2 and -1 nucleotides modified for cloning (*Nco*I site): AG → CC
pBSaraFX-ThαUTR-*saci_1116*-CtSS	pSVAaraFX-*saci_1116*-CtSS with 5'-UTR insertion from *saci_1401* (16 nt; Thα), -2 and -1 nucleotides modified for cloning (*Nco*I site): AA → CC
pBSaraFX-SlaBUTR-*saci_1116*-CtSS	pSVAaraFX-*saci_1116*-CtSS with 5'-UTR insertion from *saci_2354* (27 nt; SlaB), -2 and -1 nucleotides modified for cloning (*Nco*I site): GT → CC
pBSaraFX-SlaAUTR-*saci_1116*-CtSS	pSVAaraFX-*saci_1116*-CtSS with 5'-UTR insertion from *saci_2355* (13 nt; SlaA), -2 and -1 nucleotides modified for cloning (*Nco*I site): GT → CC
pBSaraFX-*alba*UTR-AC-*saci_1116*-CtSS	pBSaraFX-*alba*UTR-*saci_1116*-CtSS, -2 and -1 nucleotides: AC

**Name**	**Description**
pBSaraFX-*alba*UTR-AA-*saci_1116*-CtSS	pBSaraFX-*alba*UTR-*saci_1116*-CtSS, -2 and -1 nucleotides: AA
pSVAaraFX-*lacS*-CtSS	*lacS* expression plasmid based on pSVAaraFX-CtSS
pBSaraFX-*saci_0330*UTR-*lacS*-CtSS	*lacS* expression plasmid generated based on pBSaraFX-*saci_0330* UTR-*saci_1116*-CtSS
pBSaraFX-ThαUTR-*lacS*-CtSS	*lacS* expression plasmid generated based on pBSaraFX-Thα UTR-*saci_1116*-CtSS
pBSaraFX-SlaBUTR-*lacS*-CtSS	*lacS* expression plasmid generated based on pBSaraFX-SlaB UTR-*saci_1116*-CtSS
pBSaraFX-SlaAUTR-*lacS*-CtSS	*lacS* expression plasmid generated based on pBSaraFX-SlaA UTR-*saci_1116*-CtSS
pBSaraFX-*alba*UTR-*lacS*-CtSS	*lacS* expression plasmid based on pBSaraFX-*alba*UTR-*saci_1116*-CtSS
pSVAaraFX-*saci_1904*-CtSS	*saci_1904* expression plasmid based on pSVAaraFX-CtSS
pBSaraFX-*alba*UTR-*saci_1904*-CtSS	*saci_1904* expression plasmid based on pBSaraFX-*alba*UTR-CtSS
pSVAaraFX-*saci_1909*-NtSS	*saci_1909* expression plasmid based on pSVAaraFX-NtSS
pBSaraFX-*alba*UTR-*saci_1909*-NtSS	*saci_1909* expression plasmid based on pBSaraFX-*alba*UTR-NtSS
pSVAaraFX-*saci_1911*-Nt/CtSS	*saci_1911* expression plasmid based on pSVAaraFX-Nt/CtSS
pBSaraFX-*alba*UTR-*saci_1911*-Nt/CtSS	*saci_1911* expression plasmid based on pBSaraFX-*alba*UTR-Nt/CtSS
pSVAaraFX-*saci_1915*-Nt/CtSS	*saci_1915* expression plasmid based on pSVAaraFX-Nt/CtSS
pBSaraFX-*alba*UTR-*saci_1915*-Nt/CtSS	*saci_1915* expression plasmid based on pBSaraFX-*alba*UTR-Nt/CtSS
pSVAaraFX-*saci_1916*-NtSS	*saci_1916* expression plasmid based on pSVAaraFX-NtSS
pBSaraFX-*alba*UTR-*saci_1916*-NtSS	*saci_1916* expression plasmid based on pBSaraFX-*alba*UTR-NtSS
pSVAaraFX-*saci_1921*-NtSS	*saci_1921* expression plasmid based on pSVAaraFX-NtSS
pBSaraFX-*alba*UTR-*saci_1921*-NtSS	*saci_1921* expression plasmid based on pBSaraFX-*alba*UTR-NtSS
pSVAaraFX-*saci_2033*-CtSS	*saci_2033* expression plasmid based on pSVAaraFX-CtSS

**Name**	**Description**
pBSaraFX-*alba*UTR-*saci_2033*-CtSS	*saci_2033* expression plasmid based on pBSaraFX-*alba*UTR-CtSS (Schmerling et al., 2024a)
pSVAaraFX-SSO2093-NtSS	SSO2093 expression plasmid based on pSVAaraFX-NtSS
pBSaraFX-*alba*UTR-SSO2093-NtSS	SSO2093 expression plasmid based on pBSaraFX-*alba*UTR-NtSS
pSVAaraFX-*saci_1085*-NtSS	*saci_1085* expression plasmid based on pSVAaraFX-NtSS
pBSaraFX-*alba*UTR-*saci_1085*-NtSS	*saci_1085* expression plasmid based on pBSaraFX-*alba*UTR-NtSS (Schmerling et al., 2024b)
pBSaraFX-*alba*UTR-noSD-*saci_1116*-CtSS	pBSaraFX-*alba*UTR-*saci_1116*-CtSS, complete mutation of 5 nt long SD motif within *alba* 5'-UTR
pBSaraFX-*alba*UTR-8SDmut-*saci_1116*-CtSS	pBSaraFX-*alba*UTR-*saci_1116*-CtSS, 8 nt long SD motif within *alba* 5'-UTR created by targeted nucleotide mutation
pBSaraFX-*alba*UTR-8SDins-*saci_1116*-CtSS	pBSaraFX-*alba*UTR-*saci_1116*-CtSS, 8 nt long SD motif within *alba* 5'-UTR created by nucleotide insertions

Supplementary Table 2. Primers used in this study. 5'-UTR sequences are underlined. Relevant nucleotides for the *alba* 5'-UTR modification by QuikChange PCRs® are indicated by the use of bold letters.

Name	Sequence (5' → 3')	Description
**Insertion of 5'-UTR sequences into pSVAaraFX-*saci_1116*-CtSS**		
UTR-fwd	CGTATTACCGCCTTTGAGTG	Fwd-primer for the insertion of 5'-UTRs
saci_1322-UTR-rev	ATACCATGGAAACCACCTATCGGTATGATAAGTAAGACGCTTATC	Insertion of *saci_1322* (Alba) 5'-UTR, *Nco*I site
saci_0330-UTR-rev	ATACCATGGTTGGGTCACCTTTTGGTATGATAAGTAAGACGCTTATC	Insertion of *saci_0330* 5'-UTR, *Nco*I site
saci_1401-UTR-rev	ATACCATGGGAGACACCGTTTCTGGTATGATAAGTAAGACGCTTATC	Insertion of *saci_1401* (Thα) 5'-UTR, *Nco*I site
saci_2354-UTR-rev	ATACCATGGACATACACCCGATATGTTATAAATTGGTATGATAAGTAAGACGCTTATC	Insertion of *saci_2354* (SlaB) intergenic region, *Nco*I site
saci_2355-UTR-rev	ATACCATGGTTTTCACTTTCGGTATGATAAGTAAGACGCTTATC	Insertion of *saci_2355* (SlaA) 5'-UTR, *Nco*I site
**QuikChange PCR® primers for modification of *alba*UTR in pBSaraFX-*alba*UTR-*saci_1116*-CtSS**		
QC-*alba*UTR-AA-fwd	GATAGGTGGTTT**AA**ATGGCTCCTTTAGATCCAACCATAAAG	Modification of -2 and -1 nucleotides to "AA"
QC-*alba*UTR-AA-rev	GGAGCCAT**TT**AAACCACCTATCGGTATGATAAGTAAGACG	
QC-*alba*UTR-AC-fwd	GATAGGTGGTTT**AC**ATGGCTCCTTTAGATCCAACCATAAAG	Modification of -2 and -1 nucleotides to "AC"
QC-*alba*UTR-AC-rev	GGAGCCAT**GT**AAACCACCTATCGGTATGATAAGTAAGACG	
QC-noSD-fwd	GCGTCTTACTTATCATACCGAT**TCCAC**GTTTCCATGGCTCCTTTAGATC	Complete mutation of SD motif (5 nts)
QC-noSD-rev	GATCTAAAGGAGCCATGGAAAC**GTGGA**ATCGGTATGATAAGTAAGACGC	
QC-8SDmut-fwd	GCGTCTTACTTATCATACCG**TG**AGGTG**A**TTTCCATGGCTCCTTTAGATC	Mutation of *alba* 5'-UTR nucleotides for

Name	Sequence (5' → 3')	Description
QC-8SDmut-rev	GATCTAAAGGAGCCATGGAAA**T**CACCT**CA**CGGTATGATAAGTAAGACGC	establishment of 8 nt SD motif
**Generation of pBSaraFX-*alba*UTR-8SDins-*saci_1116*-CtSS** (8nt long SD motif within *alba* 5'-UTR by nucleotide insertions)		
SD-fwd	ATGTTCTTTCCTGCGTTATC	Insertion of modified *alba* 5'-UTR (8SDins) in pSVAaraFX-*saci_1116*-CtSS
8SD-ins-rev	ATACCATGGAAAC**T**CACCT**C**ATCGGTATGATAAGTAAGACGCTTATC	
**Generation of cloning vector pBSaraFX-*alba*UTR-NtSS**		
saci_1322-UTR-NtSS-fwd	GATAGGTGGTTTAAATGGCTTGGAGTCATCCACAATTTG	Insertion of *saci_1322* (*alba*) 5'-UTR in pSVAaraFX-NtSS (overlap PCR)
saci_1322-UTR-NtSS-rev	AGCCATTTAAACCACCTATCGTTATGATAAGTAAGACGCTTATC	
**Amplification of genes of interest**		
Saci_1116-*Nco*I-fwd	TAACCATGGCTCCTTTAGATCCAACCATAAAG	Amplification of *saci_1116*, addition of *Nco*I and *Xho*I sites
Saci_1116-*Xho*I-rev	TATCTCGAGAAAACTGTTGTAGAATACTCGTCTC	
lacS-SSO-*Nco*I-fwd	GATCCATGGCTTACTCATTTCCAAATAGCTTTAG	Amplification of *lacS* from *S. solfataricus* addition of *Nco*I and *Xho*I sites
lacS-SSO-*Xho*I-rev	GATCTCGAGGTGCCTTAATGGCTTTAC	
Saci_1904-*Nco*I-fwd	CGGCCATGGTGGACTTTGTCTTCTTTACAGATC	Amplification of *saci_1904*, addition of *Nco*I and *BamH*I sites
Saci_1904-*Bam*HI-rev	CGAGGATCCACTCTTGACACCACAACGTAG	
Saci_1909-*Nco*I-fwd	TATCCATGGTGCTTAGTGTTGTGATACCGGC	Amplification of *saci_1909*, addition of *Nco*I and *Xho*I sites
Saci_1909-*Xho*I-rev	TAACTCGAGGGAGTTCAGTCTATTAAATACCCAAC	
Saci_1911-*Nco*I-fwd	TATCCATGGTGTGGTCGATTGAGATCCC	Amplification of *saci_1911*, addition of *Nco*I and *Xho*I sites
Saci_1911-*Xho*I-rev	CGGCTCGAGTCCTAATAAGTAGCCTAATATG	
Saci_1915-*Nco*I-fwd	TATCCATGGTGCCCAAAGACTTCAGTG	Amplification of *saci_1915*, addition of *Nco*I and *Xho*I sites
Saci_1915-*Xho*I-rev	GCGCTCGAGACTAAACATCGAATTACTCC	
Saci_1916-*Nco*I-fwd	CTTCCATGGTGATAATGGAAAAACCGATAG	Amplification of *saci_1916*, addition of *Nco*I and *Xho*I sites
Saci_1916-*Xho*I-rev	CGTCTCGAGTACTTTCATTAAATCCTC	
Saci_1921-*Nco*I-fwd	GCGCCATGGTGCTTAGTATAACATTCCTTATTG	Amplification of *saci_1921*,

Name	Sequence (5' → 3')	Description
Saci_1921-XhoI-rev	GCGCTCGAGTATATCAACTTCAATCTGTTTC	addition of *Nco*I and *Xho*I sites
*Saci_2033-Nco*I-fwd	GAACCATGGTGGCTGAAAAATACGTGATAG	Amplification of *saci_2033*, addition of *Nco*I and *Xho*I sites
*Saci_2033-Xho*I-rev	GGTCTCGAGACCCCCAATAGTCTTAGC	
SSO2093-*Nco*I-fwd	GTACCATGGGTACGTTTGGTTATAAATTAG	Amplification of SSO2093, addition of *Nco*I and *Xho*I sites
SSO2093-*Xho*I-rev	GGACTCGAGAAGTTTATATAAAGCAAATCC	
*Saci_1085-Nco*I-fwd	GCCCGCCATGGGGTCAGAGCAGGGTCC	Amplification of *saci_1085*, addition of *Nco*I and *Xho*I sites
*Saci_1085-Xho*I-rev	CGGGCCTCGAGTCATTGTGGTTTGTCAGTAC	

Supplementary Table 3. Effect of *alba* 5'-UTR insertion as well as changes in -2 and -1 nucleotide identities on esterase reporter protein (Saci_1116-CtSS) amounts. To determine the share of Saci_1116 in the total cell protein of *S. acidocaldarius* [%], the specific activities of Saci_1116-CtSS in cell lysate samples (compare Fig. 3 in the main text) were set in relation to the specific activity of the purified enzyme (124.5 U/mg). Fold changes are calculated as mean values, comparing the amount of reporter protein in lysate samples without any 5'-UTR to the different *alba* 5'-UTR variants (i.e. -2 and -1 nucleotides: AA, AC, CC). Expression cultures were harvested at OD_{600nm} values of 0.6.

Nucleotide identity at -2 and -1 positions of *alba* 5'-UTR	Specific activity in crude lysates [U/mg]	Amount of Saci_1116 in total protein [%]	Mean value fold change (-/+UTR)
no UTR	3.4 ± 0.2	2.6 ± 0.1	-
AA	16.0 ± 2.3	12.7 ± 1.8	4.7
AC	14.4 ± 1.1	11.5 ± 0.9	4.2
CC	13.1 ± 0.8	10.4 ± 0.7	3.9

Supplementary Table 4. Selected UTR sequences from *S. acidocaldarius*. Information on the gene ID, the current annotation, the original UTR sequence in the genome of *S. acidocaldarius* as well as the adapted and inserted UTR sequence are given ("CC" at -2 and -1 positions (underlined) for maintenance of *Nco*I site in *saci_1116*-CtSS expression constructs). SD motifs are shown in bold.

Gene	Protein	Genome UTR sequence	Inserted UTR sequence
saci_0330	Unknown function	AAA**AGGTGA**CCCAAAG	AAA**AGGTGA**CCCAACC
saci_1401	Thermosome subunit α (Thα)	AGAAAC**GGTG**TCTCAA	AGAAAC**GGTG**TCTCCC
saci_2354	S-layer protein B (SlaB)	AATTTATAACATATCG**GGTG**TATGTGT	AATTTATAACATATCG**GGTG**TATGTCC
saci_2355	S-layer protein A (SlaA)	GAAA**GTGA**AAAGT	GAAA**GTGA**AAACC
saci_1322	Alba	GAT**AGGTG**GTTTAA	GAT**AGGTG**GTTTCC

Supplementary Table 5. Effect of the different 5'-UTR sequences on the protein amounts of reporter protein Saci_1116-CtSS. To determine the share of Saci_1116 in the total cell protein of *S. acidocaldarius* [%], the specific activities of Saci_1116-CtSS in cell lysate samples (compare Fig. 4 in the main text) were set in relation to the enzyme's specific activity (124.5 U/mg). Fold changes are calculated as mean values, comparing the amount of reporter enzyme in lysate samples without any 5'-UTR sequence to the amount of POI produced under the influence of a 5'-UTR. Expression cultures were harvested at OD_{600nm} values of 0.8-1.2.

5'-UTR	Specific activity in crude lysates [U/mg]	Amount of Saci_1116 in total protein [%]	Mean value fold change (-/+UTR)
no UTR	4.2 ± 0.8	3.2 ± 0.7	-
Saci_0330	14.2 ± 3.1	11.3 ± 2.5	3.4
Thα	10.5 ± 1.4	8.4 ± 1.2	2.5
SlaB	10.1 ± 1.3	8.0 ± 1.0	2.4
SlaA	9.3 ± 1.2	7.4 ± 1.0	2.2
Alba	12.3 ± 1.1	9.8 ± 0.9	3.0

Supplementary Table 6. Studies on protein production in archaea comparing the use of leadered mRNAs and leaderless mRNAs.

Host organism	5'-UTR (gene name)	5'-UTR origin	Tested 5'-UTR sequence	SD sequence length [nts]	5'-UTR GC content [%]	Reporter protein	Effect of 5'-UTR addition on reporter production (leadered mRNA vs. leaderless mRNA)	Reference
Haloferax volcanii	- (UTR1; random seq.)	- (random seq.)	UACCA CAUUU CAGGC AAGAU (20 nts)	0	40	DHFR (dihydrofolate reductase)	↑ (513%)	Brenneis et al., 2007; Hering et al., 2009
Haloferax volcanii	Seq. variation of random UTR1	-	----- CAUUU CAGGC AAGAU (15 nts)	0	40	DHFR	↓ (32%)	Hering et al., 2009
Haloferax volcanii	Seq. variation of random UTR1	-	----- ----- CAGGC AAGAU (10 nts)	0	50	DHFR	↓ (41%)	Hering et al., 2009
Haloferax volcanii	Seq. variation of random UTR1	-	UACCA CAUUU CAGGC ----- (15 nts)	0	70	DHFR	↓ (19% of protein production using leaderless transcript)	Hering et al., 2009
Haloferax volcanii	Seq. variation of random UTR1	-	UACCA CAUUU ----- ----- (10 nts)	0	30	DHFR	↓ (73%)	Hering et al., 2009
Haloferax volcanii	- (UTR2; random seq.)	-	CGACUCACAACUG GACUUCA (20 nts)	0	50	DHFR	↑ (230%)	Brenneis et al., 2007
Haloferax volcanii	- (UTR3; random seq.)	-	ACAACGGCCUCUC GAUACCA (20 nts)	0	55	DHFR	↓ (51%)	Brenneis et al., 2007
Haloferax volcanii	- (UTR4; random seq.)	-	GCUGCAGCAGAGA ACUUGGC (20 nts)	0	60	DHFR	→ (119%)	Brenneis et al., 2007

**Host organism**	**5'-UTR (gene name)**	**5'-UTR origin**	**Tested 5'-UTR sequence**	**SD sequence length [nts]**	**5'-UTR GC content [%]**	**Reporter protein**	**Effect of 5'-UTR addition on reporter production (leadered mRNA vs. leaderless mRNA)**	**Reference**
Haloferax volcanii	*hlr* (HVO_2837; hoxA like transcription-nal regulator)	*Haloferax volcanii*	AGAUAGCGAGA CAG (14 nts)	0	50	DHFR	↓ (56%)	Brenneis & Soppa, 2009
Haloferax volcanii	*hp* (HVO_0721; conserved hypothetical protein)	*Haloferax volcanii*	GACCACGACGACG CGGGUCGAU (22 nts)	0	68	DHFR	↓ (60%)	Brenneis & Soppa, 2009
Haloferax volcanii	*gvpF* (gas vesicle formation; *gvpFGHIJKL M*)	*Halobac-terium salinarum*	169 nts	6 (3 conse-cutive)		mGFP6 (green fluorescent protein for haloarchaea)	↓ (OD_{600} 1.2: ↓ (35%))	Born & Pfeifer, 2019; Sartorius-Neef & Pfeifer, 2004
Haloferax volcanii	*gvpD* (gas vesicle formation; operon *gvpDE*)	*Halobac-terium salinarum*	ACAGUCGUGAAGC GAAGAAAGCCUCA CCUACUAGUCGGG AGUGCUCUACCGC CAUCGACU**GGAG**A GA**A**GUA (71 nts)	5 (4 conse-cutive)		mGFP6	↓ (6-26%); value decreasing with increasing OD_{600} value; OD_{600} 1.2: ↓ (10%)	Born & Pfeifer, 2019; Sartorius-Neef & Pfeifer, 2004; Jones et al., 1989
Haloferax volcanii	*gvpA* (gas vesicle formation; operon *gvpACNO*)	*Halobac-terium salinarum*	GGGUUAAUCC CAGAUCACCA (20 nts)	0		mGFP6	growth phase-dependent; OD_{600} 0.6: ↓ (41%) OD_{600} 1.2: → (100%)	Born & Pfeifer, 2019; Sartorius-Neef & Pfeifer, 2004; DasSarma et al., 1987

**Host organism**	**5'-UTR (gene name)**	**5'-UTR origin**	**Tested 5'-UTR sequence**	**SD sequence length [nts]**	**5'-UTR GC content [%]**	**Reporter protein**	**Effect of 5'-UTR addition on reporter production (leadered mRNA vs. leaderless mRNA)**	**Reference**
Sulfolobus acido-caldarius	*saci_0330* (hypoth. protein)	*Sulfolobus acidocal-darius*	AAA**AGGTGA**CCCA ACC (16 nts)	6	50	Saci_1116-CtSS	↑ (340%)	This study
Sulfolobus acido-caldarius	*saci_1401* (thermosome α subunit)	*Sulfolobus acidocal-darius*	AGAAAC**GGUG**UCU CCC (16 nts)	4	56	Saci_1116-CtSS	↑ (250%)	This study
Sulfolobus acido-caldarius	*saci_2354* (S-layer protein B)	*Sulfolobus acidocal-darius*	AAUUUAUAACAUA**U** CG**GGUG**UAUGUCC (27 nts)	4	33	Saci_1116-CtSS	↑ (240%)	This study
Sulfolobus acido-caldarius	*saci_2355* (S-layer protein A)	*Sulfolobus acidocal-darius*	GA**A**A**GUGA**AAACC (13 nts)	4	38	Saci_1116-CtSS	↑ (220%)	This study
Sulfolobus acido-caldarius	*saci_1322* (Alba)	*Sulfolobus acidocal-darius*	GAU**AGGUG**GUUU CC (14 nts)	5	50	Saci_1116-CtSS	↑ (300%; OD_{600} 0.8-1.2) ↑ (390%; OD_{600} 0.6)	This study
Haloferax volcanii	*hsp70* (heat shock protein 70)	*Natrinema* sp. J7	CACG (4 nts)	0	75	BgaH (β-galactosidase)	↓ (44%)	Chen et al., 2015
Haloferax volcanii	*hsp70*, -2 nucleotide variation: C→G	*Natrinema* sp. J7	CAGG (4 nts)	0	75	BgaH	↓ (0%)	Chen et al., 2015
Haloferax volcanii	*hsp70*, -2 nucleotide variation: C→A	*Natrinema* sp. J7	CAAG (4 nts)	0	50	BgaH	↓ (75% of protein production using leaderless transcript) (172% of native 5'-UTR)	Chen et al., 2015

Host organism	5'-UTR (gene name)	5'-UTR origin	Tested 5'-UTR sequence	SD sequence length [nts]	5'-UTR GC content [%]	Reporter protein	Effect of 5'-UTR addition on reporter production (leadered mRNA vs. leaderless mRNA)	Reference
Haloferax volcanii	*hsp70*, -2 nucleotide variation: C→U	*Natrinema* sp. J7	CAUG (4 nts)	0	50	BgaH	↑ (239% comp. to leaderless transcript) (% of native 5'-UTR)	Chen et al., 2015
Haloferax volcanii	*hsp70*, -1 nucleotide variation: G→C	*Natrinema* sp. J7	CACC (4 nts)	0	75	BgaH	↑ (166% comp. to leaderless transcript) (% of native 5'-UTR)	Chen et al., 2015
Haloferax volcanii	*hsp70*, -1 nucleotide variation: G→A	*Natrinema* sp. J7	CACA (4 nts)	0	50	BgaH	↓ (64% comp. to leaderless transcript) (% of native 5'-UTR)	Chen et al., 2015
Haloferax volcanii	*hsp70*, -1 nucleotide variation: G→U	*Natrinema* sp. J7	CACU (4 nts)	0	50	BgaH	→ (116% comp. to leaderless transcript) (% of native 5'-UTR)	Chen et al., 2015

**Host organism**	**5'-UTR (gene name)**	**5'-UTR origin**	**Tested 5'-UTR sequence**	**SD sequence length [nts]**	**5'-UTR GC content [%]**	**Reporter protein**	**Effect of 5'-UTR addition on reporter production (leadered mRNA vs. leaderless mRNA)**	**Reference**
Haloferax volcanii	Artificial seq., partially with *gvpH* 5'-UTR (involved in gas vesicle formation)	*Halobac-terium salinarum*	AUGCCCAUGGACA GA UGA UCGAU **CGCAU** **GGAGGUCA** **AGAAAUA** (43 nts; 10 nts derived from *fdx* promoter (ferredoxin); spacing of 13 nts)<br><br>**Bold: *gvpH* 5'-UTR sequence (20 nts).**<br><br>SD mutation: 2 or 4 nts of SD motif; or full SD.<br><br>Spacer length variation.<br>Native spacer: 7 nts.<br>Tested: 1, 4, 5, 10 nts spacer	7 (6 conse-cutive)	47<br><br>Full SD mutation: 44	GvpH(15 nts)-BgaH (β-galactosidase)	Native UTR: 100%<br>Leaderless mRNA ("ΔSD"): **no protein**.<br><br>Comparison of SD motif mutation to native SD:<br>↓ (5-50% SD mutation (2/4 nts))<br><br>↓ (20% full SD motif mutation)<br><br>↓ (0% for 1 nt spacer)<br>↓ (10% for 5 nt spacer)<br>→ (100% for 4 and 10 nt spacers)	Sartorius-Neef & Pfeifer, 2004

Supplementary Table 7. Studies comparing protein production in archaea with various 5'-UTR modifications, such as truncations or extentions, mutations of the SD motif, or the replacement of a native 5'-UTR by other 5'-UTR sequences. SD motifs are underlined. Mutation sites are marked with a red color.

**Host organism**	**5'-UTR (gene name)**	**5'-UTR origin**	**Tested 5'-UTR sequence**	**SD sequence length [nts]**	**5'-UTR GC content [%]**	**Reporter protein**	**Effect of 5'-UTR alterations**	**Reference**
Haloferax volcanii	Artificial seq., partially with *gvpG* 5'-UTR (involved in gas vesicle formation)	*Halobac-terium salinarum*	AUGCCCAUGG **CGCGGAACAGC AACAA GGAGG CCGAUAAUGC** (41 nts; 10 nts derived from *fdx* promoter (ferredoxin))<br>**Bold: *gvpG* 5'-UTR sequence.**<br><br>5'-UTR alteration (length + SD seq. motif)	5	59	**GvpG**-(GvpH) (immuno-detection)	5'-UTR alteration (reduced length + SD seq. motif changed to 4 nts (3 consecutive)): ↓	Sartorius-Neef & Pfeifer, 2004
Haloferax volcanii	*sod2* SD motif variants (HVO_2913; superoxide dismutase)	*Haloferax volcanii*	Native: GAUAC GGAGGUUA CACAUU (19 nts)<br><br>SD motif mutations: instead of A:C; instead of G,C,U:A<br><br>Exemplary SD mutants:<br><br>No SD: GAUAC AACAAAAC CACAUU<br><br>7 nt SD: GAUAC AGAGGUGA CACAUU<br><br>8 nt SD: GAUAC GGAGGUGA CACAUU	Native: 7 (6 conse-cutive);<br>SD mutagenesis study	Native: 42<br><br>No SD: 32	Sod2(90 nts)-DHFR (489 nts)	For all SD mutant variants: no SD-7 nt SD variant: → same transl. efficiencies<br><br>8 nt SD: ↓ signif. lower transl. efficiency	Kramer et al., 2014

Host organism	5'-UTR (gene name)	5'-UTR origin	Tested 5'-UTR sequence	SD sequence length [nts]	5'-UTR GC content [%]	Reporter protein	Effect of 5'-UTR alterations	Reference
Thermo-coccus kodaka-rensis	*gdh* (from P_{gdh}) (glutamate dehydro-genase)	*Thermo-coccus kodaka-rensis*	ACAGGUGGUAUGA (13 nts)<br><br>SD motif mutation site in red.	6<br><br>Mutation of 2nd nt in SD motif	46	RpoL (subunit L of archaeal DNA-dependent RNA polyme-rase)-HA	Mutation of 2nd nt in SD motif:<br>G → C: →<br>G → A: ↓ (approx. 15%)<br>G → U: ↓ (approx. 50%)	Santangelo et al., 2008
S. solfa-taricus in vitro transla-tional system	SD mutants of ORF104 SD (putative ribosomal protein; bicistronic with ORF143)	*Saccharo-lobus solfatari-cus*	…UGAGGUGA… (7 nts spacer, AUG)<br>…UGACGUCA…	8 → disruption (3 conse-cutive)		ORF104 (-ORF143); ^{32}P-labelled oligonucleotide probe	↓ (0%)<br>Translational block upon SD motif disruption.<br><br>Leaderless construct: translation; lower than with native 5'-UTR with SD	Condó et al., 1999
S. solfa-taricus in vitro transla-tional system	SD mutants of ORF143 SD (putative NUSA-like termination/ anti-termination transcription factor; bicistronic with ORF104)	*Saccharo-lobus solfatari-cus*	…GAGGUGA… (8 nts)<br>…GACGUCA…<br><br>Note: GUG start codon of ORF143 is immediately consecutive to ORF104 stop codon (UGA), preceded by the SD motif located within ORF104	7 → disruption (2 conse-cutive)		(ORF104-) ORF143; ^{32}P-labelled oligonucleotide probe	↓ (0%)<br>Translational block upon SD motif disruption	Condó et al., 1999
Sulfolobus islandicus	Artificial seq. *araS* promoter modific.: SD insertion (AG GUGAAG)	-	Extension from GAGCAU (6 nts) to GAG**AAUGAGGUGA AGCU**CAU (20 nts)	8	Short UTR: 50 Exten-ded UTR: 45	LacS-CtHis$_6$ (*S. solfataricus*)	↑ (280%)	Peng et al., 2012

**Host organism**	**5'-UTR (gene name)**	**5'-UTR origin**	**Tested 5'-UTR sequence**	**SD sequence length [nts]**	**5'-UTR GC content [%]**	**Reporter protein**	**Effect of 5'-UTR alterations**	**Reference**
Methanococcus maripaludis	*pst* (inorganic phosphate (Pi)-specific transport system)<br><br>in comparison to:<br><br>*slp* (S-layer protein)<br>*hmmA* (histone A)<br>*mcrB* (methyl coenzyme M reductase)	*Methanococcus maripaludis*	5'-UTR sequences adapted for *NdeI* site for cloning (-3 to -1 nucleotides).<br><br>UTR-*pst*:<br>AUAAACCU**GGGAGGUG**UCUCAU (22 nts)<br><br>UTR-*slp*:<br>AUAAAAAAAGUAACACAACAA**AGGUGA**AUUCAU (33 nts)<br><br>UTR-*hmmA*:<br>AUAAAAGAUU**GAGGUG**AUCAU (21 nts)<br><br>UTR-*mcrB*:<br>AUAUAUAUCAAAAAAAU**AGGAGUGG**UUCAU (30 nts)	UTR-*pst*: 8<br>UTR-*slp*: 6<br>UTR-*hmmA*: 6<br>UTR-*mcrB*: 8	UTR-*pst*: 45<br>UTR-*slp*: 24<br>UTR-*hmmA*: 28<br>UTR-*mcrB*: 23<br><br>→ 5'-UTRs *slp, hmmA, mcrB*: lower GC content, very GC-poor besides SD motifs	mCherry	Exchange of native 5'-UTR by 5'-UTR of highly expressed genes:<br><br>↑ Signif. more reporter fluorescence under low (inducing) and high phosphate conditions.<br><br>UTR-*pst* vs. UTR-*slp*: ↑ (approx. 170%, low Pi conc.)<br><br>UTR-*pst* vs. UTR-*hmmA*: ↑ (approx. 250%, low Pi conc.)<br><br>UTR-*pst* vs. UTR-*mcrB*: ↑ (approx. 250%, low Pi conc.)<br><br>Spacer length variation of UTR-*pst* from 6 to 5 or 4 nts: ↑	Akinyemi et al., 2021

References Supplementary Material

Akinyemi, T.S., Shao, N., Lyu, Z., Drake, I.J., Liu, Y., and Whitman, W.B. (2021). Tuning gene expression by phosphate in the methanogenic archaeon *Methanococcus maripaludis*. ACS Synth Biol 10(11), 3028-3039. doi: 10.1021/acssynbio.1c00322.

Born, J., and Pfeifer, F. (2019). Improved GFP variants to study gene expression in Haloarchaea. Front Microbiol 10, 1200. doi: 10.3389/fmicb.2019.01200.

Brenneis, M., Hering, O., Lange, C., and Soppa, J. (2007). Experimental characterization of *cis*-acting elements important for translation and transcription in halophilic archaea. PLoS Genet 3(12), e229. doi: 10.1371/journal.pgen.0030229.

Brenneis, M., and Soppa, J. (2009). Regulation of translation in haloarchaea: 5'- and 3'-UTRs are essential and have to functionally interact *in vivo*. PLoS One 4(2), e4484. doi: 10.1371/journal.pone.0004484.

Chen W., Yang, G., He, Y., Zhang, S., Chen, H., Shen, P., Chen, X., and Huang, Y.-P. (2015). Nucleotides flanking the start codon in *hsp70* mRNAs with very short 5'-UTRs greatly affect gene expression in Haloarchaea. PLoS ONE 10(9), e0138473. doi: 10.1371/journal.pone.0138473.

Condó, I., Clammaruconi, A., Benelli, D., Ruggero, D., and Londei, P. (1999). *Cis*-acting signals controlling translational initiation in the thermophilic archaeon *Sulfolobus solfataricus*. Mol Microbiol 34(2), 377-384. doi: 10.1046/j.1365-2958.1999.01615.x.

DasSarma, S., Damerval, T., Jones, J.G, and Tandeau de Marsac, N. (1987). A plasmid-encoded gas vesicle protein gene in a halophilic archaebacterium. Mol Microbiol 1(3), 365-370. doi: 10.1111/j.1365-2958.1987.tb01943.x.

Hering, O., Brenneis, M., Beer, J., Suess, B., and Soppa, J. (2009). A novel mechanism for translation initiation operates in haloarchaea. Mol Microbiol 71(6), 1451-1463. doi: 10.1111/j.1365-2958.2009.06615.x.

Jones, J.G., Hackett, N.R., Halladay, J.T., Scothorn, D.J., Yang, C.F., Ng, W.L., and DasSarma, S. (1989). Analysis of insertion mutants reveals two new genes in the pNRC100 gas vesicle gene cluster of *Halobacterium halobium*. Nucleic Acids Res 17(19), 7785-7793. doi: 10.1093/nar/17.19.7785.

Kramer, P., Gabel, K., Pfeiffer, F., and Soppa, J. (2014). *Haloferax volcanii*, a prokaryotic species that does not use the Shine Dalgarno mechanism for translation initiation at 5'-UTRs. PLoS One 9(4), e94979. doi: 10.1371/journal.pone.0094979.

Lipps, G. (2004). The replication protein of the *Sulfolobus islandicus* plasmid pRN1. Biochem Soc Trans 32(2), 240-244. doi: 10.1042/bst0320240.

Peng, N., Deng, L., Mei, Y., Jiang, D., Hu, Y., Awayez, M., et al. (2012). A synthetic arabinose-inducible promoter confers high levels of recombinant protein expression in hyperthermophilic archaeon *Sulfolobus islandicus*. Appl Environ Microbiol 78(16), 5630-5637. doi: 10.1128/AEM.00855-12.

Santangelo, T.J., Cubonova, L., and Reeve, J.N. (2008). Shuttle vector expression in *Thermococcus kodakaraensis*: contributions of *cis* elements to protein synthesis in a hyperthermophilic archaeon. Appl Environ Microbiol 74(10), 3099-3104. doi: 10.1128/AEM.00305-08.

Sartorius-Neef, S., and Pfeifer, F. (2004). *In vivo* studies on putative Shine-Dalgarno sequences of the halophilic archaeon *Halobacterium salinarum*. Mol Microbiol 51(2), 579-588. doi: 10.1046/j.1365-2958.2003.03858.x.

Schmerling, C., Schroeder, C., Zhou, X., Busche, T., Kalinowski, J., Montero, L., et al. (2024a). Glycerol degradation in the thermoacidophilic crenarchaeon *Sulfolobus acidocaldarius* involves an unusual glycerol-3-phosphate dehydrogenase. bioRxiv preprint. doi: 10.1101/2024.02.29.582781

Schmerling, C., Zhou, X., Goers, P. E., Koestlbacher, S., Kessenbrock, T., et al. (2024b). *De novo* synthesis of fatty acids in Archaea via an archaeal fatty acid synthase complex. bioRxiv preprint. doi: 10.1101/2024.07.05.601840

van der Kolk, N., Wagner, A., Wagner, M., Wassmer, B., Siebers, B., and Albers, S.V. (2020). Identification of XylR, the activator of arabinose/xylose inducible regulon in *Sulfolobus acidocaldarius* and its application for homologous protein expression. Front Microbiol 11, 1066. doi: 10.3389/fmicb.2020.01066.
